# Design, Synthesis and Architectures of Hybrid Nanomaterials for Therapy and Diagnosis Applications

**DOI:** 10.3390/polym10050527

**Published:** 2018-05-14

**Authors:** Micaela A. Macchione, Catalina Biglione, Miriam Strumia

**Affiliations:** 1 Departamento de Química Orgánica, Facultad de Ciencias Químicas, Universidad Nacional de Córdoba, Av. Haya de la Torre esq. Av. Medina Allende, Córdoba X5000HUA, Argentina; mmacchione@fcq.unc.edu.ar; 2 Instituto de Investigación y Desarrollo en Ingeniería de Procesos y Química Aplicada (IPQA), CONICET. Av. Velez Sárfield 1611, Córdoba X5000HUA, Argentina; 3 Institut für Chemie und Biochemie, Freie Universität Berlin, Takustr. 3, 14195 Berlin, Germany; catabiglione@zedat.fu-berlin.de

**Keywords:** hybrid nanomaterials, inorganic-polymeric, nano-architectures

## Abstract

Hybrid nanomaterials based on inorganic nanoparticles and polymers are highly interesting structures since they combine synergistically the advantageous physical-chemical properties of both inorganic and polymeric components, providing superior functionality to the final material. These unique properties motivate the intensive study of these materials from a multidisciplinary view with the aim of finding novel applications in technological and biomedical fields. Choosing a specific synthetic methodology that allows for control over the surface composition and its architecture, enables not only the examination of the structure/property relationships, but, more importantly, the design of more efficient nanodevices for therapy and diagnosis in nanomedicine. The current review categorizes hybrid nanomaterials into three types of architectures: core-brush, hybrid nanogels, and core-shell. We focus on the analysis of the synthetic approaches that lead to the formation of each type of architecture. Furthermore, most recent advances in therapy and diagnosis applications and some inherent challenges of these materials are herein reviewed.

## 1. Introduction

The diverse pathophysiological conditions of diseases lead to a continuous demand for acceptable pharmacokinetic products and thus, novel therapeutic approaches are required. In recent years, parallel to the ongoing development of conventional treatment strategies, nanometer-sized structures in the scale range of 50 to 500 nm [1,2], where this upper limit is still subject of continuous discussion and updating, have demonstrated their full potential to become the best candidates for innovative therapeutic and diagnosis approaches. Consequently, the design and synthesis of highly specific smart nanomaterials have been the main concern of many academic and industrial researchers worldwide. Therefore, rapid advances are being made in the application of nanotechnology in medicine and pharmaceutics for a specific diagnostic or therapeutic purpose [3]. The design of functional smart nanomaterials requires the rational planning of the final product considering its relation between structure and properties. Choosing the building blocks and combining them into an either ordered or random architecture using an appropriate synthetic strategy are the essential steps in an efficient design process. Finally, as the physicochemical properties of the resulting material will modulate different interactions with the environment, it is indispensable to have a deep knowledge of these properties to optimize their technological applications and especially biomedical ones.

In this context, the strategy of nanoarchitectonics for construction of functional materials looks promising because it deals with well-defined sequential processes and controls in assembling nano-components on the basis of chemical and physical mechanisms [4]. The concept of nanoarchitectonics is based on the dynamical development of functional materials by concerted harmonization of various effects and interactions to accomplish material organization and the stimulation of spontaneous processes [5,6]. This requires various techniques and phenomena including control at atomic or molecular levels, fabrication based on chemical reactions, structural control based on application of various physical stimuli, and self-assembly/organization [7]. In this way, the creation of reliable nanomaterials or nanosystems is possible considering that the main players are not the individual nano-parts but their interactions, which cause unexpected functionalities to emerge [8]. These new functionalities can be especially valuable for biomedical applications.

The use of polymeric materials has attracted attention in the development of therapy and diagnosis devices because they can be designed as multifunctional platforms responsive to the physical and chemical stimuli of the biological environment, their physical-chemical properties can be adapted through synthesis, and they can be easily modified to attach different kinds of biomolecules, for example, targeting molecules, specific proteins or antibodies complementary to receptors highly expressed on the desired target tissues [9,10]. Synthetic polymers can be obtained with great uniformity, accurate molecular weight, controlled topology, and precisely selected end groups [11]. Moreover, the combination of polymeric materials with, for instance, dendrimers [12,13,14], liposomes [15,16], or inorganic nanoparticles (INPs) such as silicon dioxide nanoparticles [17], magnetic nanoparticles [18], plasmonic nanoparticles [19], carbon nanotubes [20], carbon dots [21], fullerene [22] or others, gives hybrid nanomaterials with additional and extraordinary properties. For the case of INPs, examples of these enhanced properties are the magnetic behavior obtained by combining magnetic nanoparticles with polymer matrices, or the more reliably protection of drug cargo displayed by mesoporous silica/polymer nanoparticles compared with purely organic nanomaterials [23]. Besides, the inorganic phase increases the mechanical strength, and thermal stability, allows to modulate the refractive index, and favors the rheological properties of the final material, depending on its shapes and sizes [2].

Some kinds of INPs can undergo aggregation when they are directly exposed to the biological system [24,25]. By coating them with appropriate polymers, the colloidal stability of the nanodevices can be increased, the activation of the immune system can be avoided, cytotoxicity can be reduced and circulation time in the blood stream can be prolonged [24,26,27]. Therefore, some polymer coatings such as polyethylene glycol (PEG) improve the biocompatibility and solubility of the inorganic nanosystems [1,28]. Thus, organic–inorganic hybrid nanomaterials are the focus of many investigations because they combine synergistically the advantageous physical-chemical properties of both the inorganic and polymeric component [2,29].

For the design of nanostructures with novel functionalities, the prediction of structure/property relationships from a multidisciplinary approach is essential [30]. The management and control of the structure/property relationship would give the basis not only of the advantageous properties but also of the limiting factors of these materials in biomedical applications. The interaction of these materials with biological systems is highly sophisticated and dependent on their structure and functional organization in the nanoscale. Therefore, before this can become a clinical reality, the applications of nanoparticles must be carefully evaluated [31,32].

Some of the main requirements that nanoparticles must fulfill for their use in medical applications are low cytotoxicity and high biocompatibility, a good colloidal stability in physiological conditions, resistance to chemical degradation, improved blood circulation time, reduction of the non-specific distribution through the incorporation of targeting moieties, easy and controlled loading of therapeutic agents, and efficient release of the cargo inside cells [33,34,35]. In this way, the targeted drug delivery is one of the most intensively explored areas of research, since the targeted therapies can dramatically improve the therapeutic efficacy of classical treatments by using specific ligands such as monoclonal antibodies, lectins, peptides, hormones, vitamins, nucleotides or drugs in order to increase doses at the disease site and reduce side effects [3,33].

**Figure 1:**
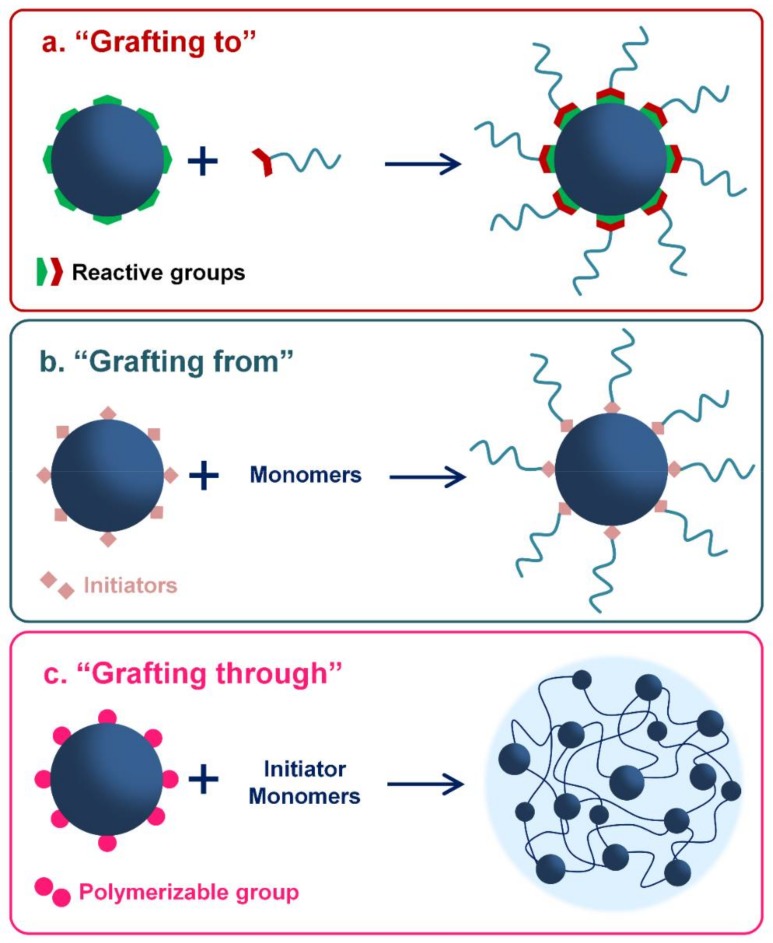
Strategies of polymer grafting: (**a**) grafting to, (**b**) grafting from and (**c**) grafting through.

One of the most common approaches used for the synthesis of this kind of hybrid nanomaterial is based on the physical adsorption of the polymer onto the surface of the INPs [3]. On the other hand, hybrid materials with covalent interactions between the polymer and the INP can be achieved by “grafting-to”, “grafting-from” or “grafting-through” approaches (Figure 1). Specifically, these last three synthetic pathways are more laborious than simple physical adsorption but allow a better control of the chemical modification, mainly when specific molecules or functional groups and size distribution are required.

Besides, covalent grafting seems to be safer for medical applications because it does not exhibit the risk of desorption of the inorganic nanoparticle in the physiological environment [36]. Firstly, “grafting-to” consists in synthetizing nanoparticles and polymers individually and connecting them; the end functionalized polymer reacts with reactive sites on the nanoparticle’s surface [37,38]. In the “grafting-from” method, the polymer chains grow in situ from an initiator that has been previously anchored to the nanoparticle’ surface [37,39,40]. Finally, in the “grafting-through” approach, the polymerizable groups are anchored onto the surface of nanoparticles. Then, the polymerization is started in the solution that contains initiator, monomers, and modified NPs, which act as crosslinker agent. Therefore, copolymerization of both monomers takes place and the inorganic phase is incorporated inside polymer chains [37,38].

**Figure 2:**
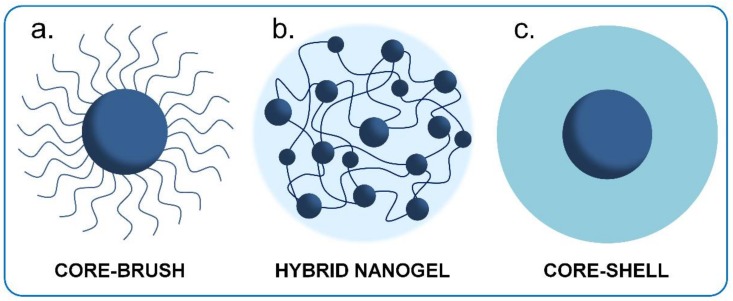
Different architectures of hybrid nanomaterials: (**a**) core-brush, (**b**) hybrid nanogel, and (**c**) core-shell.

This updated review focuses on those hybrid nanostructures that present core-brush (Figure 2a), hybrid nanogel (Figure 2b) and core-shell (Figure 2c) architectures, because we believe that they have the greatest potential for application in therapy and diagnosis for nanomedicine. In the first type, core-brush architecture, independent polymeric chains with controlled chain length are tethered to the surface of INPs. In hybrid nanogels, INPs are incorporated, physical or covalently, inside of a crosslinked polymer matrix. In the third type, core-shell architecture, a compact polymeric shell is bonded to the surface of INPs.

The content of this review is divided between the above described nano-architectures. First, we will analyze the synthetic routes that lead to the formation of each architecture and the strategies for the control of structural parameters in each case. Subsequently, we will highlight examples of recent research that illustrate their potential in therapy and diagnosis applications from the controlled release of drugs to diagnostic imaging techniques. We will analyze those properties directly associated with the architecture and discuss about structure/properties relationships to achieve a better understanding of these hybrid materials. Emphasis will be placed on several representative types of INPs with biomedical applications, such as magnetic nanoparticles (MNPs) as magnetite and maghemite nanoparticles (Fe_3_O_4_NPs and Fe_2_O_3_NPs, respectively), silica nanoparticles (SiO_2_NPs), mesoporous silica nanoparticles (MSNPs), gold nanoparticles (AuNPs), and carbon nanoparticles (CNPs).

## 2. Core-Brush Nanoparticles

Nanostructures consisting in an inorganic core and polymer brushes are one of the most studied conformations among hybrid nanomaterials. The term “polymer brush” is used to denote the extended conformation of individual not crosslinked polymer chains directly attached by one chain-end to a solid support when grafted at a sufficiently high density [41,42,43] (Figure 2a). These polymeric chains help to stabilize INPs dispersions and allow one to modulate the physicochemical properties of the final nanostructure to synthetize materials with new functionalities [44]. For biological applications, covalent brush-shells seem to be the best choice compared to physically grafted systems, due to the risks of desorption with this latter method [36].

Covalent binding of the polymer chains can be achieved via a “grafting-from” or “grafting-to” method, which were explained above. The “grafting-from” approach for the synthesis of core-brush nanostructures consists in surface-initiated reversible-deactivation radical polymerization (SI-RDRP). Reversible-deactivation radical polymerization means that the propagating radicals are deactivated reversibly and, consequently, the equilibrium between the remaining active radicals and dormant species is established [45]. In SI-RDRP, all polymer chains are grown simultaneously and directly attached to the surface, resulting in high grafting densities with precise control over the structural parameters of the tethered polymer chains as polymer architecture, composition, molecular weight, and brush thickness, etc. [43]. In addition, SI-RDRP can be applied to a wide range of monomers and substrates.

Otherwise, synthetic routes that involve the covalent link of end-functionalized polymer chains to a reactive surface are considered to be “grafting-to” approach for core-brush nanostructures. Within this approach, condensation and “click” chemistry reactions are the most used. Condensation reactions are those in which two molecules react to form the addition product and a small molecule like water. “Click” chemistry reactions correspond to an efficient and selective type of reactions. Among them, the most employed have been the Cu(I)-catalyzed and orthogonal strain-promoted azide–alkyne cycloaddition, the Diels–Alder cycloaddition, the azide–nitrile cycloaddition, and the thiol-ene reaction [46,47,48,49,50,51,52].

### 2.1. Grafting-from Approaches for the Synthesis of Core-Brush Nanoparticles

One of the approaches for the synthesis of inorganic–organic core-brush morphologies is grafting-from, where the formation of polymer brushes is produced from the surface of inorganic particles. SI-RDRP has proven to be the most powerful tool to prepare core-brush inorganic-polymeric nanomaterials. In this review, we will discuss the most used strategies for core-brush synthesis, which are surface-initiated atom transfer radical polymerization (SI-ATRP) and surface-initiated reversible addition–fragmentation chain transfer polymerization (SI-RAFT), to show the main features of core-brush grafted-from systems. A complete review of the synthetic routes employed for grafting-from polymer brushes was recently presented by Giussi et al. [53].

#### 2.1.1. Atom Transfer Radical Polymerization (ATRP)

**Figure 3:**
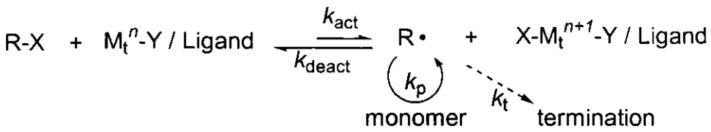
Scheme of atom transfer radical polymerization (ATRP) equilibrium. Reprinted with permission from ref. [56]. © 2012, Wiley Online Library.

ATRP is a robust and powerful technique for grafting anchored polymer brushes which allows one to evenly and precisely control the chemistry of the injection, size, functionalities, and architecture of the polymers as well as to reach the uniform growth of all the chains [54,55]. The mechanism by which the polymerization is produced by ATRP is schematized in Figure 3.

ATRP is controlled by equilibrium between propagating radicals and dormant species, predominately in the form of initiating alkyl halides/macromolecular species (R-X) [54,56]. The radicals, or the active species, are generated through a reversible redox process catalyzed by a transition metal complex M_t_^n^-Y/L, whereby M_t_^n^ represents the kind of a transition metal with oxidation state n, L is the ligand and Y may be another ligand, which undergoes a one-electron oxidation with concomitant abstraction of the halogen atom, X, from a dormant species, R-X (alkyl halide) [11,57]. Therefore, radicals that have an intermittent growth (R•) and a deactivating agent that is the transition metal complex in its highest oxidation state (X-M_t_^n+1^-Y/L) are produced [54]. The propagation occurs by the addition of the radical species to the monomer with a constant of propagation (kp), similarly to conventional radical polymerization [57]. The deactivating agent reacts with the radical to re-form the dormant species and the activator [57,58]. The chain growth continues until the terminal propagating radical reduces metallic oxidized species. Therefore, the mechanism is controlled by a balance between the radicals that spread and the dormant species in which the halides of alkyl (R-X) that initiate the radical species are the predominant species.

ATRP can be mediated by many redox-active transition-metal complexes among which the redox couple Cu(I)/L and X-Cu(II)/L is the most frequently used. The rate of ATRP depends on several factors as the rate constant of propagation, the concentrations of monomer and growing radicals, the nature of the ligand and monomer/dormant species and reaction conditions, etc. [54].

SI-ATRP is the predominant SI-RDRP method used for the synthesis of polymer brushes onto the surface of INPs through the grafting-from approach [59]. Typically, in SI-ATRP, an ATRP initiator is tethered onto the surface prior to polymerization. Then, since the chains grow from the surface, the rate of propagation could be limited due to the need for diffusion of the monomer to the chain ends, which affects the polymerization kinetics [59].

Silane chemistry has been applied to metal oxide surfaces to promote surface-polymer adhesions [60]. Thus, for Fe_3_O_4_NPs and SiO_2_NPs, a silane with a halogen terminal group can be used as an ATRP initiator. These molecules can be covalently attached to the nanoparticle surface by a silanization reaction, which means the reaction between the surface accessible hydroxyl groups on the INP and a silyl-anchoring group. The most used halogen-ended silanization agents for SI-ATRP are shown in Table 1.

Another approach consists in linking 3-aminopropyltriethoxysilane (APTES) and then, through a condensation reaction, obtaining a stable and tethered ATRP initiator. As an example, this reaction was performed using 3-chloropropionic acid [60,68], 2-bromoisobutyryl bromide [69,70], 2-bromo-2-methylpropionyl bromide [71], bromoacetyl bromide [72], among others. 3-chloropropionic acid was also introduced through a ligand exchange with the oleic acid on the surface of the pristine nanoparticles [73]. A different route to attach an ATRP initiator to the substrate is to modify the surface with 3-(trimethoxysilyl)propyl methacrylate (MPS) in order to introduce a vinyl group for further polymerization with a halogen-ended vinyl monomer, as 4-vinylbenzyl chloride, to finally achieve an ATRP initiator shell onto the INPs [74].

SI-ATRP has been applied and allow one to have precise control over the particle brush architecture (grafting density, molecular weight, molecular weight distribution, connectivity, and composition) with monomers as diverse as hydrophilic methacrylate monomers (2-(*N*-morpholino)-ethyl methacrylate [61], oligo(ethylene glycol) methacrylate [61], 2-sulfoethyl methacrylate [61], hydroxyethyl methacrylate [61], glycidyl methacrylate [61], styrene [64], (ethylene glycol) methacrylate and methyl methacrylate [60], (methacrylic acid) and tert-butylmetharylate [75], itaconic acid and poly(ethylene glycol)methyl acrylate [76], glycidyl methacrylate [72], *N*-isopropylacrylamide [77], etc.

##### Smart Hybrid Core-Brush Nanomaterials by ATRP

Monomers confer the physicochemical properties to the polymer matrix. Indeed, some monomers can introduce a smart behavior in response to external stimuli. When an external stimulus is applied to smart polymers, a change in their properties takes place, for instance, a change in conformation, in solubility, alteration of the hydrophilic/hydrophobic balance or release of a bioactive molecule (e.g., drug molecule). This also includes a combination of several responses at the same time. The most important stimuli are pH, temperature, ionic strength, light, and redox potential [19,20].

Thermo-responsive polymers are some of the most widely investigated smart polymers because of their potential biomedical applications and easiness to control the stimulus [78]. Thermo-responsive polymer brushes can modulate the catalytic activity of enzymes. Marten et al. [67] produced the covalent immobilization of porcine pancreas trypsin within the thermo-responsive brushes grafted-Fe_3_O_4_MNPs. The catalytic activity of trypsin was strongly increased when the particles were heated across the transition temperature of the polymer shell, an effect that was attributed to the shell shrinkage.

Other highly important smart materials that were also synthetized by SI-ATRP are the pH-sensitive materials. The application and development of pH-responsive systems has been extensively studied for stimuli-responsive drug delivery. For instance, poly(2-(diethylamino)ethyl methacrylate) (PDEAEMA) brushes can act as a good gatekeeper of MSNPs to control access to the pores via a pH-dependent open-close mechanism. Thus, this core-brush system allows a well-controlled release of rhodamine B (RhB) from the mesopores through adjusting pH of the solution [71].

**Figure 4:**
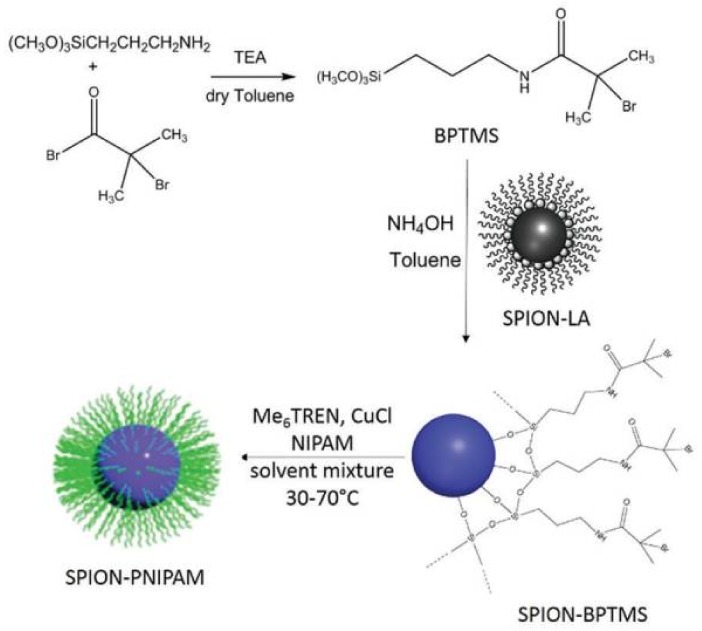
Scheme of SPION-PNIPAM nanoparticles by ATRP. Reprinted with permission from ref. [80]. © 2018, Royal Society of Chemistry.

Dually responsive core-brush systems as thermo and pH responsive are also challenging from the synthetic point of view. As an example, thermo and pH dual-responsive core-brush systems developed by ATRP were the Janus structures synthetized by Isojima et al. [79]. The nanodevice consists of Fe_3_O_4_MNPs coated uniformly with a pH-dependent and temperature-independent polymer (poly(acrylic acid), PAA) shell that was functionalized further by the asymmetric attachment of a second polymer to one side of the surface coating. The second polymer used was either pH-independent polystyrene sodium sulfonate or temperature-dependent poly(*N*-isopropylacrylamide) (PNIPAM). In addition, Wu et al. [65] reported the synthesis of well-defined thermo and pH dual-responsive poly(*N*-isopropylacrylamide)-b-poly(4-vinylpyridine)-grafted silica nanoparticles (SiO_2_NPs-g-PNIPAM-b-P4VP). In this study, swelling of the diblock copolymer chains due to change in pH was observed whereas this behavior was not observed while varying the temperature of the environment. However, the hydrophilic-hydrophobic phase transition of the inner PNIPAM block was probed by in situ variable-temperature 1H NMR spectroscopy. The authors proposed that the shrinkage of the inner thermo-responsive PNIPAM chains was compensated by the stretching of the flexible outer protonated P4VP chains. These results show that the combination of a pH-responsive with a thermo-responsive polymer can further alter the hydrophilic/hydrophobic balance. Other dually responsive core-brush nanocarriers with pH-dependent and temperature-sensitive release characteristics are the smart theranostic nanoparticles recently synthetized by Yar et al. [80]. In this study, no cytotoxic superparamagnetic iron oxide nanoparticles (SPIONs) coated with PNIPAM by SI-ATRP demonstrated an excellent doxorubicin (DOX) loading efficiency (96 wt % in PBS buffer), which is highly desirable for a drug delivery vehicle (Figure 4). The DOX release studies revealed pH and temperature-dependence, which were not reported for PNIPAM before. Lastly, these nanoparticles could generate contrast in magnetic resonance imaging (MRI), acting as a potential SPION-based contrast agent. This MRI modality combined with the dually responsive controlled drug release behavior makes these nanoparticles valuable theranostic candidates.

**Figure 5:**
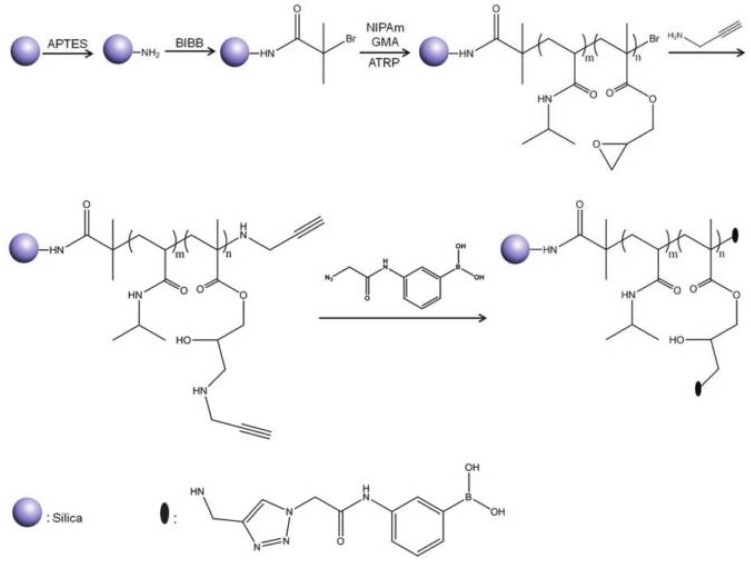
Scheme of the synthesis of SiO_2_NPs@poly(NIPAM-co-GMA)@APBA particles by combining SI-ATRP with the CuAAC “click” reaction. Reprinted with permission from ref. [70].

Jiang et al. [70] developed a temperature and pH-responsive boronate affinity material for effective separation of glycoproteins composed of silica cores and flexible polymer brushes, denoted as SiO_2_NPs@poly(NIPAM-co-GMA)@APBA, where GMA means glycidyl methacrylate and APBA means 3-(2-azidoacetyl-amino)phenylboronic acid (Figure 5). Boronic acid ligand-functionalized materials have shown great potential for selective recognition, immobilization, and enrichment of biomolecules containing a cis-diol structure, for example, glycoproteins. The capture/release process could be easily controlled by switching the pH value as in mildly basic aqueous media. Boronic acids could covalently bind the cis-diol moiety to generate cyclic esters, while under acidic conditions the boronate ester bond could be hydrolyzed. SiO_2_NPs@poly(NIPAM-co-GMA) were synthetized by SI-ATRP and then azide-functionalized APBA was introduced into the polymer brushes through a Cu(I)-catalyzed azide-alkyne cycloaddition (CuAAC) “click” reaction. The resulting nanohybrid exhibited excellent specificity and affinity towards glycoproteins because of the high density of boronic acid ligands on the polymer brushes. Moreover, glycoprotein binding on the nanohybrid could be controlled by varying the pH of the binding buffer.

Polymer brushes improve INPs stability, biocompatibility, and toxicity in physiological medium [3]. For instance, a recent study showed that poly((ethylene glycol) methacrylate-b-methyl methacrylate) P(PEGMA-b-MMA) block copolymers graft on SPIONs had higher ability for resisting proteins than that of uncoated Fe_3_O_4_NPs [60]. P(PEGMA) brush was also reported to prevent recognition by macrophages and therefore increased the in vivo circulation time of the hybrid materials [81]. Cytotoxicity of P(PEGMA)-grafted Fe_3_O_4_NPs core-brush nanoparticles with well-controlled properties was evaluated in macrophage cells and 3T3 fibroblasts [73]. No significant cytotoxicity was observed. A MRI of these nanoparticles in water confirmed its contrast enhancement effect in T2-weighted sequences so that P(PEGMA)-grafted nanoparticles may be a good candidate as a T2-contrast agent [73].

**Figure 6:**
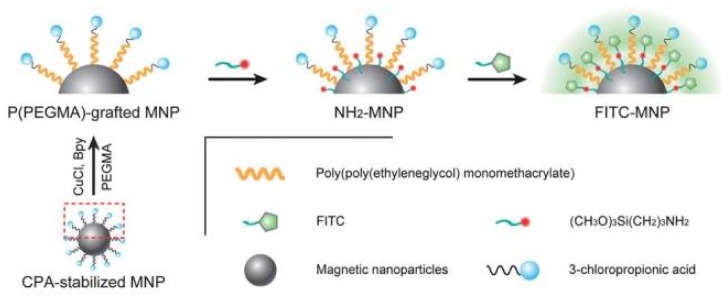
Scheme of the synthetic route of well-dispersed bifunctional nanoparticles (FITC-MNPs). Reprinted with permission from ref. [68]. © 2012, Royal Society of Chemistry.

Also, core-brush systems with bifunctional nanoparticles with magnetism and fluorescence are of particular importance because of their broad range of potential applications, especially in vitro and in vivo bioimaging, biological labeling, and biomedicine. Lu et al. [68] produced water-soluble P(PEGMA)-grafted Fe_3_O_4_NPs to which fluorescein isothiocyanate (FITC) was covalently attached, obtaining both fluorescent imaging and a MRI probe (Figure 6). In this case, these nanostructures, referred to as FITC-MNPs, also presented low cytotoxic effect in 3T3 fibroblasts. The uptake of the FITC-MNPs by macrophage cells was found similar to the P(PEGMA)-MNPs, which indicated the good biocompatibility of the P(PEGMA) chains on the FITC-MNP surface was little influenced by further introducing FITC groups. The FITC-MNPs can be efficiently up taken by breast cancer cells, which might be due to the high stability of the P(PEGMA) chains in the cell membranes. Their optical and magnetic properties, biocompatibility, cytotoxicity, and cellular imaging make them excellent candidates for multi-bioapplications.

Another case of fluorescent/magnetic nanoparticles is the hybrid nanodevice based on an iron oxide core, fluorescent dye-sensitized silica shell fluorescein isothiocyanate (DySiO_2_-FITC), and P(PEGMA) brushes [74]. The Fe_3_O_4_NPs/DySiO_2_-g-P(PEGMA) nanoparticles have undergone more extensive endocytosis by the 3T3 fibroblasts than the pristine Fe_3_O_4_NPs/DySiO_2_ core-shell nanoparticles without the grafted of P(PEGMA) brushes, showing their improved biocompatibility.

Fluorescent/magnetic nanoparticles were also obtained by grafting a fluorescent monomer 9-(4-vinylbenzyl)-9H-carbazole from MNPs via iron(III)-mediated ATRP with activators generated by electron transfer (AGET ATRP) [69]. Well-dispersed bifunctional nanoparticles (Fe_3_O_4_NPs@PVBK

b-P(PEGMA)) in water were obtained via consecutive AGET ATRP of hydrophilic monomer P(PEGMA). Once more, P(PEGMA) polymer chains improve the nanoparticles’ stability in water. The Fe_3_O_4_NPs@PVBK-b-P(PEGMA nanoparticles showed an effective imaging ability by enhancing the negative contrast in MRI.

Recently, numerous multi-responsive drug delivery nanocarriers have been designed to improve the therapeutic efficacy and minimize collateral effects. These nanocarriers are better candidates for controlling the drug delivery and release process in the complicated blood circulation and pathological environment compared with single-stimulus-responsive nanocarriers. Besides, more efficient effect is expected against tumor cells. An efficient drug nanocarrier with pH, reduction, and light triple-responsive drug release capability based on hollow MSNPs coated with PDEAEMA, was produced by Zhang et al. via SI-ATRP [82]. High loading capacity of DOX and quick release were obtained. Results have shown that the nanocarriers were efficiently internalized by HeLa cancer cells and presented enhanced release of DOX into the cytoplasm under external UV light irradiation [82].

In addition, core-brush nanostructures can also be functionalized to perform “active targeting” against tumors. “Active targeting” refers to specific interactions between a drug carrier and the target cells, usually through specific ligand-receptor interactions [1]. Folic acid, for example, has been recognized as a highly selective and effective ligand for the active targeting of cancer cells, because the folate receptor is normally overexpressed only on cancer cells, and restricted in normal tissues. Huang et al. [81] used SI-ATRP to graft SPIONs with P(GMA-co-PEGMA). Then the GMA groups were conjugated to the folic acid, via “click” chemistry in order to develop a nanostructure with the ability of performing the specific targeting and detection of tumor cells in bioimaging, biodetection, and other in vivo medical applications.

##### Metal-Free ATRP Approaches

SI-ATRP allows one to control the film thickness, graft density, and chain length with low polydispersity. Moreover, ATRP is known to be versatile and easy to perform (mild conditions) [36]. However, one of the strongest disadvantages of ATRP is the use of metal catalysts, which presumes a limitation for biomedical applications [36]. Recently, several ATRP techniques using reduced or null catalyst complex concentrations have emerged, opening new possibilities for the synthesis of polymeric materials for therapy and diagnosis. As an example, enzymatic polymerization was presented as a green biocatalytic approach without toxic residues in polymers after reaction and, hence, with great potential in the production of materials for food processing and biomedical applications. Recently, Zhou et al. [23] employed peroxidase mimetic catalytic ATRP to synthetize poly(*N*,*N*-dimethylaminoethyl methacrylate) (PDMAEMA) brushes-grafted MSNPs in a facile and highly efficient manner. The release of rhodamine 6G (Rh6G) out of MSNPs was studied at different pH. In acidic environments, the electrostatic repulsion caused by the positively charged tertiary ammonium of PDMAEMA induced the stretching out of polymer brushes on MSNP surfaces, which allowed the diffusion of Rh6G out of the mesopores of MSNPs. Otherwise, surface-initiated electrochemically mediated atom transfer radical polymerization has been used recently by Matyjaszewski´s group [44]. Well-defined dense block copolymer brushes with the desired composition from SiO_2_NPs were prepared under constant potential electrolysis conditions. Results show that the rate of polymerization could be enhanced by applying more negative potentials, which resulted in an increase in grafting density.

#### 2.1.2. Reversible Addition–Fragmentation Chain Transfer (RAFT)

RAFT polymerization is another reversible-deactivation radical polymerization. To achieve its deactivation−activation equilibrium, a chain transfer agent (CTA) is employed. Transfer agents for addition–fragmentation mechanism are unsaturated compounds of general structure Z-C(X)-A-R. The double bond C=X is reactive towards radical addition [83]. X can be either CH_2_ or S and Z is a group that confers the transfer agent with an appropriate reactivity towards propagating radicals. The intermediate radicals are also stabilized by Z group [83]. R is a homolytic leaving group to form R•, which should be able to efficiently re-initiate polymerization [83].

**Figure 7:**
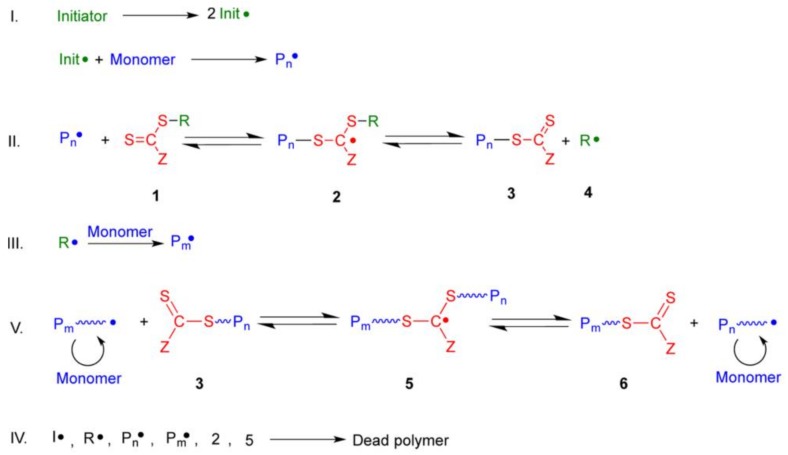
Mechanism of reversible addition–fragmentation chain Transfer (RAFT) polymerization. Reprinted with permission from ref. [84]. © 2015, American Chemical Society.

As ATRP, an external initiator is also required for RAFT mechanism (Figure 7), with initiation proceeding as in conventional radical polymerization. Therefore, the initiator decomposes to form two fragments (Init•) that react with a single monomer molecule to produce a propagating polymeric radical, denoted P_n_• [84]. The propagating radical P_n_• adds to the CTA (thiocarbonylthio compound) (1) to form the radical intermediate (2), which subsequently fragments to give another thiocarbonylthio group (3) and a new radical (R•) (4) [84]. The radical (R•) may then react with the newly formed thiocarbonyl group or reinitiate polymerization by reacting with monomer to form a new propagating radical (P_m_•) [84].

The present radicals are “shared” among all species that have not yet undergone termination (P_n_• and S=C(Z)S-P_n_) and the main equilibrium of activation−deactivation is established by degenerative chain transfer between propagating (P_m_ or P_n_) and dormant chains (3/6) [84]. Given the fast initialization and a rapid equilibrium between active radical-containing chains and dormant thiocarbonylthio-containing chains, an equal opportunity for growth is provided, which leads to polymers with narrow molecular weight distributions [84,85]. Chains in their active form react via bi-radical termination to form chains that cannot grow further.

In SI-ATRP, as the initiator is anchored to the surface, the polymer chains are also tethered to the surface. However in SI-RAFT polymerization, since the initiator is a separate moiety from the CTA, grafted polymeric chains can be obtained either by tethering the initiator or the CTA agent to the substrate, and in the latter case, either the Z-group [86] or the R-group [87,88,89] of the RAFT agent can be used as an anchor. In the first approach, the initiator is attached to the solid surface and CTA RAFT agent is in the solution in order to achieve the production of radicals directly at the surface and at the CTA RAFT agent. On the contrary, when the CTA agent is tethered to the NPs surface the polymerization is initiated by a free initiator in solution [90].

When the CTA RAFT agent is fixed to the surface through its stabilizing Z-group, CTA RAFT agent will be tied to the solid surface during the whole polymerization and located close to the surface, so the chain growth is discouraged because of sterical hindrance. Otherwise, if the reinitiating R-group is the attached group, the CTA RAFT agent will depart from the surface during polymerization and consequently, the sterical hindrance is lower compared with Z-group approach. Therefore, even though both types of CTA attachment approaches results in well-defined grafted polymers with monomodal molecular weight distributions, Z-group results in higher grafting density and molecular weights than those obtained in R-group [91,92]. Z-group approach is sometimes described as a grafting-to approach rather than the typical grafting-from approach because the propagating polymeric chain needs to diffuse to the surface of the particle to undergo degenerative transfer [41,90].

Like ATRP, RAFT also leads to good control over the molecular weight of the graft polymer giving hybrid particles with high uniformity and high dispersibility [93]. Silane coupling agents having RAFT moiety can be easily attached to INPs as silica and magnetite [93,94]. SI-RAFT polymerization can also employ different monomers like styrene and methyl acrylate [95]; methyl methacrylate and *N*-butyl acrylate [93]; 4-vinylpyridine [87]; and *N*-isopropylacrylamide [88], etc.

##### Smart Hybrid Core-Brush Nanomaterials by RAFT

**Figure 8:**
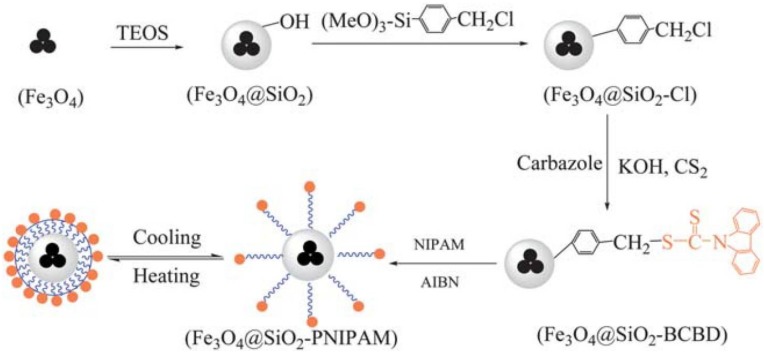
Synthetic route of thermo-responsive Fe_3_O_4_@SiO_2_NPs. Reprinted with permission from ref. [88]. © 2011, Royal Society of Chemistry.

Smart and multifunctional nanostructures can also be synthetized by RAFT approach. Water-soluble trifunctional hybrid NPs with thermo, magnetic, and fluorescent-behavior, Fe_3_O_4_@SiO_2_-PNIPAM, were prepared by Li et al. [88] via SI-RAFT polymerization, using fluorescent RAFT agent-functionalized magnetic silica NPs as the CTA and NIPAM as the monomer. The fluorescent RAFT agent, benzyl 9H-carbazole-9-carbodithioate, was synthesized on the surface of Fe_3_O_4_@SiO_2_NPs. This RAFT agent has a carbazole group as the Z group and benzyl group tethered on Fe_3_O_4_@SiO_2_NPs that was used as the R group in order to grow the thermo-responsive PNIPAM chains from the NPs (Figure 8). Furthermore, the imaging ability of this core-brush system was tested confirming an effective enhancement of the negative contrast in MRI.

Jiao et al. [96] demonstrated the importance of CTA coverage on the kinetics of RAFT polymerization. In this study, the polymerization of styrene with CTA-functionalized Fe_3_O_4_NPs in the presence of free CTAs was performed. At a high grafting density of RAFT agent (1.95 RAFT agents/nm^2^), surface-initiated chains grew longer than the coexisting free chains in solution. These results may indicate free CTAs did not play a role in the chain transfer exchange on the particles and the grafted chains grew as in free radical polymerization, which yielded polymeric chains with a higher molecular weight than the free chains in solution. Thus, radical transfer and exchange reactions were inefficient between grafts and free polymer and converted the SI-RAFT mechanism to a free radical polymerization.

Brushes onto SiO_2_NPs can be also synthetized by SI-RAFT. The synthesis of monodisperse particles with a silica core and a P(4-vinylbenzyl chloride-co-pyren-1-ylmethyl acrylate) (VBC-co-PyAc) shell using thermally autoinitiated RAFT polymerization was described by Moraes et al. [97]. In this case, the incorporation of a co-monomer (PyAc) in the SI-RAFT polymerization resulted in fluorescent particles, which were further modified with triethylamine to obtain positively charged hydrophilic particles. The resulting NPs were capable of being taken up into the endosome of human colon cancer cells because of their interaction with the negatively charged cell membrane. The stability in the media and the persistence of the fluorescence indicated the suitability of the particles for imaging the cells in vitro using confocal microscopy. Poly(acrylic acid) brushes grafted from the surface of SiO_2_NPs@PAAs by SI-RAFT resulted in good candidates for ultra-high streptavidin immobilization by virtue of its rich carboxyl groups and spherical brush structure [89].

### 2.2. Grafting-to Approaches for the Synthesis of Core-Brush Nanoparticles

Another strategy to obtain core-brush nano-architectures is first the synthesis of the polymer and then the linkage to the surface of the INP via grafting-to approach [38]. Currently, there are several reports of the synthesis of core-brush nanostructures by this approach.

Polymer chains can be synthetized using mainly ATRP [98], RAFT [99], ring-opening polymerization [100,101], among others. After polymerization, the polymer chains are covalently linked to the INP. For this purpose, the variety of reactions that can be used so far is very extensive. As an example, NIPAM can be polymerized via ATRP and then by subsequent amidation of acid-terminated polymers with 6-nitrodopamine yielded the polymeric dispersants that were grafted by ligand exchange onto oleic acid coated MNPs [102]. In the grafting-to approach, natural polymers can also be used. Popat et al. produced a chitosan coating on MSNPs via the phosphoramidate covalent bonding between phosphonate groups on the surface of the MSNPs and amino groups of chitosan [103].

Regarding AuNPs, polymer chains can be covalently anchored to the surface by means of gold-thiolate linkage. This strategy is mostly used in the synthesis of gold-core-brush NPs. For example, a disulphide bond in the middle of the polymer chain can be achieved performing ATRP with a disulphide initiator. Using this approach, thermo-responsive polymers (PNIPAM and a P(NIPAM-co-acrylamide) copolymer) were anchored to gold nanocages for controlled release with near-infrared light [104]. PNIPAM grafted to AuNPs can be also achieved by the synthesis of PNIPAM by RAFT polymerization using 2-(dodecylthiocarbonothioylthio)-2-methylpropionic acid as the transfer agent. Therefore, the resultant thiol moieties can be used to graft the polymer onto the surface of AuNPs [105].

The synthesis of nanoparticles decorated with PEG chains has been widely used for the incorporation of anticancer drugs, among them, DOX. To achieve this goal, polyethylene glycol has been modified chemically by different approaches. For instance, MNPs can be modified with a polymeric layer based on PEG-COOH due to the interaction between the hydroxyl groups in the surface of the MNP and the acid group. In this sense, Guarda et al. [106] reported a one-pot synthesis of water-soluble iron oxide nanocrystals where they coated the NPs with gallic acid-PEG via in situ ligand exchange. The nanocrystals showed higher values of specific absorption rate demonstrating the potential of the devices as nano-heaters for hyperthermia treatment. Likewise, MNPs coated with a layer composed of PEG/polyethyleneimine/polysorbate 80 were reported. The PEG oxidized during the synthesis of the MNPs results in PEG-COOH that could interact with the surface of the NPs. DOX was successfully incorporated. In vivo studies showed that these nanoparticles completely suppressed glioma growth after 28 days from the time of treatment due to the combined effect of the alternating magnetic field and DOX [107,108]. Also, MSNPs can be PEGylated on the outer surface via a mercapto-reactive maleimide-PEG linker. In this case, the inner surface of the MSNPs was modified with aza-dibenzocy-clooctyne derivatives to enable copper-free “click” chemistry reactions for the conjugation of azide-terminated cargos [109]. Another example is the PEGylation of AuNPs via grafting-to approach, which can be performed using thiolated PEG molecules [110]. Using this synthetic route, Urries et al. [111] have synthesized PEGylated magneto plasmonic NPs with a hollow or semi-hollow interior. For this purpose, amino functionalized silica-magnetic core-shell NPs were synthesized and then, AuNPs were attached to the amino groups located on the surface to grow a continuous gold shell. Finally, NPs were decorated with PEG-SH moieties and to obtain the hollow interior, selective silica etching was carried out. This hollow interior structure allows the incorporation of drugs or other interesting molecules. The resulting NPs maintain the magnetic and optical properties of the original core SPIONs and Au shells, respectively. Therefore, these NPs has potential as a theranostic platform, combining two therapeutic possibilities (drug delivery and NIR hyperthermia) and imaging capabilities in MRI applications. In addition, Sun et al. [112] synthetized highly dispersed ultrafine PEG-ylated MNPs. First, the surface of MNPs surface was modified with APTES to form amine-terminated nanoparticles. Then, NPs were grafted with PEG by introducing PEG-diacid into the reaction mixture where the amine-terminated MNPs reacted with PEG-diacid to form carboxylic acid-terminated PEG on the MNPs. Another synthetic step was performed in order to provide amine terminal groups on the PEG chain which enabled MNP/PEG conjugation with chlorotoxin (model targeting agent) and Cy5.5 (a near-infrared fluorescent dye). Finally, the tumor-targeting ability and multifunctionality of these PEG-ylated iron oxide NPs were evaluated for both MR and optical imaging in vitro and in vivo.

**Figure 9:**
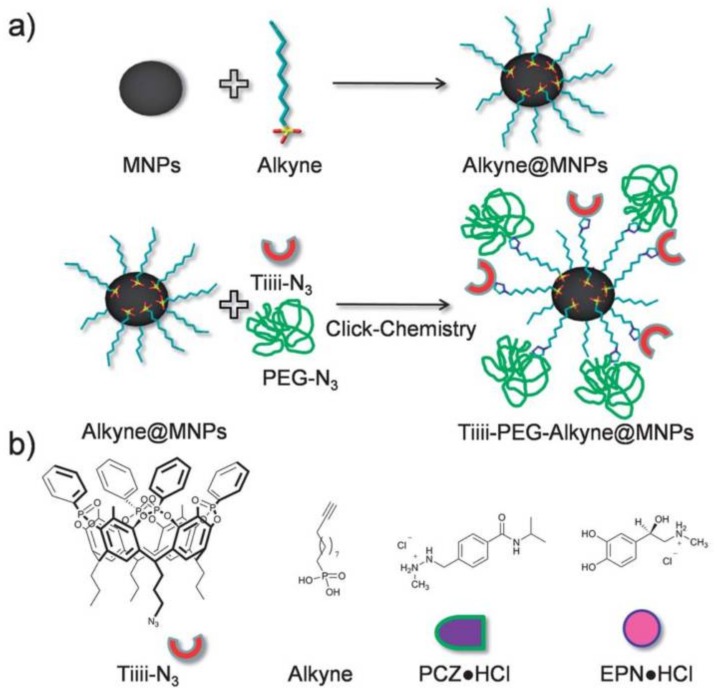
(**a**) Reaction steps for the preparation of functionalized-MNPs. (**b**) Structure of Tiiii receptors, phosphonic acid grafting agent, and the drugs used in their complexed Reprinted with permission from ref. [114]. © 2013, Royal Society of Chemistry.

Among other techniques, “click” chemistry has been widely used in core-brush nanoparticle synthesis [47]. For example, polystyrene and poly(4-vinylpyridine) were grafted to SiO_2_NPs via Cu(I)-catalyzed azide-alkyne Huisgen cycloaddition (CuAAC). To achieve this, azide-terminated polystyrene and poly(4-vinylpyridine) were synthesized and then the polymers were anchored onto alkyne-modified SiO_2_NPs by CuAAC [113]. Magnetic core-brush nanoparticles were also synthetized by “click” chemistry [114,115,116,117,118,119]. Tudisco et al. [114] reported MNPs that were surface modified with phosphate containing alkyne terminals for subsequent covalent binding via “click” chemistry of PEG chains and Tiiii receptors (Figure 9). These systems can load different bioactive molecules such as procarbazine hydrochloride (antitumor drug) and epinephrine hydrochloride (neurotransmitter) and release them as free bases. In addition, the cytotoxicity test showed that the core-brush NPs could be employed in the field of medicine due to the presence of the PEG chains.

**Figure 10:**
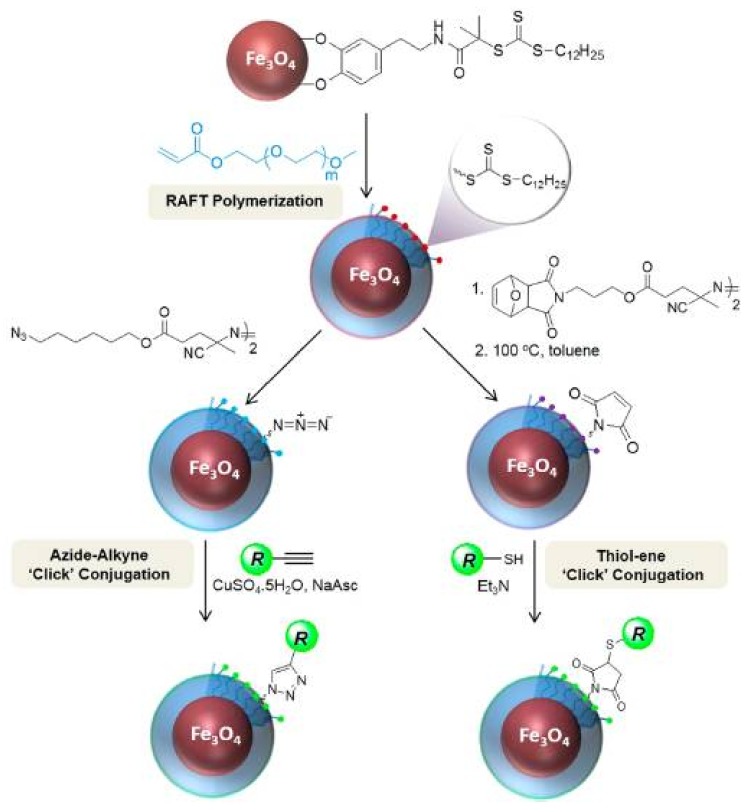
Synthesis and post-modification of polymer-coated MNPs. Reprinted with permission from ref. [119]. © 2015, American Chemical Society.

Similarly, Das et al. [115] designed a new PEG-silane with azide terminals that could easily self-assemble on the surface of the metal oxide nanoparticles through silane anchoring and simultaneously facilitate orthogonal bio-functionalization of alkyne-folate through “click” chemistry (Figure 10). They found the successful immobilization of folic acid on the NPs through this silane. The modified nanoparticle showed ability to accumulate in cellular lysosomes and mitochondrias and its circulating time was increased due to the PEG chains. Likewise, Oz et al. [119] prepared core-brush nanoparticles by coating first the MNPs with poly((ethylene glycol) methyl ether acrylate) (PEGMEA) and then modifying them with azide or thiol reactive maleimide moieties. Afterwards, the NPs were modified via “click” chemistry with different dyes. These nanodevices proved the potential use of MNPs as imaging probes due to the nature of the MNPs and the surface functionalities, which allowed targeting and optical imaging. In addition, they demonstrated that the presence of a polymeric brush-shell enhanced the dispersibility in biological media.

As mentioned previously, multi-responsive drug delivery systems have also been synthetized by grafting-to approach. Hegazy et al. [120] have recently reported a magnetic, reductive, and thermo multi-responsive nanocarrier based on core-brush magnetic mesoporous silica nanoparticles modified with PNIPAM. First, Fe_3_O_4_NPs were coated with a mesoporous silica matrix. 3-mercaptopropyl-trimethoxysilane was attached to provide SH groups on the surface of the particles and, after the disproportionation reaction between sulfhydryl group and pyridine group of S-(2-aminoethylthio)-2-thiopyridine hydrochloride), the disulfide bond (S-S) and amine group were tethered to the surface. The thermo-responsive polymer was synthesized by photoinduced electron/energy transfer-RAFT polymerization. Finally, the *N*-hydroxysuccinimide ester at the end of the PNIPAM and the primary amine groups on the surface of the MSNPs were linked covalently. DOX was easily encapsulated into the nanocarriers with a high loading capacity and quickly released in response to the stimuli of reducing agent, magnetic or hyperthermia.

## 3. Hybrid Nanogels

In recent decades, nanogels (NGs) have been widely investigated as nanodevices in the field of medicine due to their unique properties. A NG is a three-dimensional-crosslinked polymeric matrix that has nanometric sizes in its three dimensions. In this sense, these nanomaterials combine the characteristics of hydrogels and nanoparticles. The polymer network is formed by a chain with different repetitive units (monomers) that are crosslinked by a crosslinking agent [78,121,122,123,124,125,126]. The concept of hybrid nanogels refers to those in which INPs are incorporated in the crosslinked polymer matrix [127,128,129]. These nanodevices have gained considerable attention in recent years due to their exclusive properties resulting of synergistically combining both the characteristics of the nanogel and those of the inorganic nanoparticle, which gives different advantages over conventional drugs and agents in the area of optical detection, diagnosis, imaging, and drug delivery [130,131,132].

Therefore, different nanoparticles have been used for the synthesis of hybrid NGs: MNPs [125], AuNPs [133], SiO_2_NPs [129], quantum dots [134], graphene [135], carbon dots [136], silver nanoparticles [123,137], etc. Depending on the nature of the INP, different functionalities can be achieved, which allows its use as a contrast agent, in guided therapy, for photothermal treatment or hyperthermia [129].

One of the main advantages of these systems is that the porous structure of the nanogel allows the incorporation of a drug or other bioactive molecules as well as acting as a protection against the possible degradations that occur before reaching the target tissue [138]. Thus, drugs or bioactive molecules can be released by regulating the time and dose required [139]. The incorporation of INPs in a matrix provides an additional advantage to this drug delivery system: the nature of the incorporated nanoparticle can regulate the type of stimulus that can be used to release the drug in the desired conditions, which enhances the supply in different parts of the body and allows the transport of hydrophobic drugs or obtainment of smart systems [133]. Several stimuli can be used as triggers such as magnetic field, near infrared irradiation, pH, temperature, or others. In addition, in some cases incorporating NPs like mesoporous silica may improve the loading capacity of drugs.

**Figure 11:**
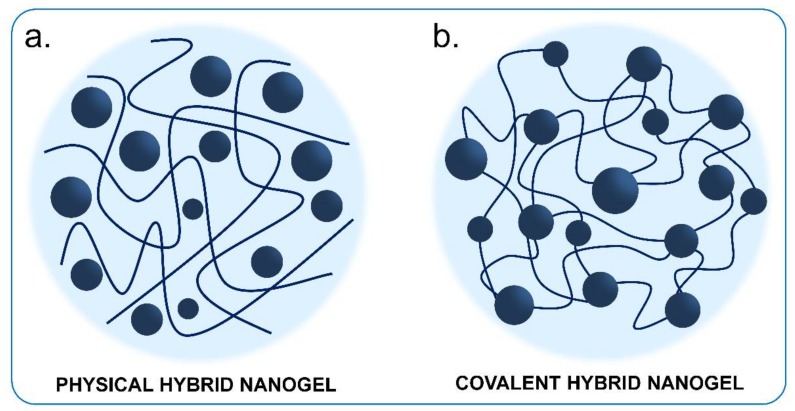
Scheme of physical (**a**) and covalent (**b**) hybrid nanogels.

INPs can be incorporated in the polymer matrix either physically or covalently (Figure 11). In the first case, NPs can be physically introduced into the matrix before or after gelation to be trapped in the NG [127,139]. We will refer to the resulting NGs as “physical hybrid nanogels” (PHNGs). Otherwise, INPs can be also covalently incorporated giving “covalent hybrid nanogels” (CHNGs) [127,139]. This last strategy is promising because it has the advantage that the nanoparticles are covalently bound and do not diffuse through the polymer matrix [140,141].

### 3.1. Physical Hybrid Nanogels

Most of the reported hybrid nanogels have been synthetized by physically incorporating the INPs. In general, PHNGs can be achieved by using different monomers, such as NIPAM [140,142,143], *N*-vinylcaprolactam [144], acrylamide (AAm) [145], chitosan [146,147] and, above mentioned different INPs.

Within this wide variety of INPs used in PHNGs, carbon nanoparticles (CNPs) are a very special type because of their excellent properties as theranostic agents. Multifunctional hybrid nanogels based on fluorescent CNPs immobilized into P(NIPAM-AAm) nanogels were prepared by one-pot free radical precipitation copolymerization of NIPAM and AAm in the aqueous dispersion of CNPs. In this case, the immobilization of CNPs takes place via hydrogen bonding interactions between polymer chains and CNPs hydroxyl/carboxyl groups. P(NIPAM-AAm) matrix provides high colloidal stability and thermo-responsive properties and the immobilized CNPs in the interior of the gel network provide the hybrid nanogels with bright, stable, and up conversion photoluminescence properties and photothermal conversion ability of near-infrared light. Besides, the resulting hybrid nanogel has a high loading capacity for a highly hydrophobic anticancer drug like curcumin. The drug release rate can be efficiently controlled by changing the temperature of local environmental media or the exogenous irradiation with near-infrared light because CNPs can convert the near-infrared light to heat [148].

The synthetic procedures to give PHNGs can be split between those that perform the polymerization of the organic matrix in the presence of pre-formed INPs, those which make the physical mixture between polymer matrix and INPs after the polymerization is completed, and those which synthetize the polymeric matrix and the INPs in situ [127].

**Figure 12:**
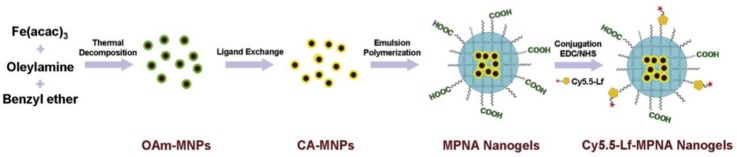
Synthetic approach for obtaining Cy5.5-Lf-MPNA nanogels (NGs). Reprinted with permission from ref. [150]. © 2013, Elsevier.

The first approach for the synthesis of PHNGs consists in the formation of the polymer matrix in a suspension of pre-formed INPs and monomer solution. This approach was used for developing thermo-responsive magnetic NGs by Liu et al. [149]. They synthesized a hybrid NG with responsiveness to temperature, magnetism, and near-infrared light that consisted of PNIPAM matrix and Fe_3_O_4_NPs. Similarly, Jiang et al. [150] reported a pH/temperature responsive magnetic nanocarrier. Firstly, oleylamine-coated Fe_3_O_4_NPs were synthesized and coated with citric acid via a ligand exchange reaction. Then, magnetic NGs were prepared by performing an emulsion polymerization of NIPAM, *N*,*N*′-methylene diacrylamide and acrylic acid (AA) in the presence of citric acid-coated Fe_3_O_4_NPs. These NGs were conjugated with Cy5.5-labeled lactoferrin (Cy5.5-Lf-MPNA) and tested as a bifunctional contrast agent for both MRI and optical imaging for glioma diagnosis (Figure 12). These NGs change in size and have hydrophilic/hydrophobic properties at different in vivo environments, circulate in blood for a longer time compared with bared MNPs functionalized with Cy5.5-labeled lactoferrin, and can specifically accumulate in glioma tissues through the combination of the active targeting ability of lactoferrin and the enhanced passive targeting ability provided by the pH/temperature sensitivities of the nanogels. Finally, in vitro and in vivo tests indicated that the MRI/fluorescence imaging of glioma was realized with high sensitivity and specificity by the application of Cy5.5-Lf-MPNA NGs [150].

**Figure 13:**
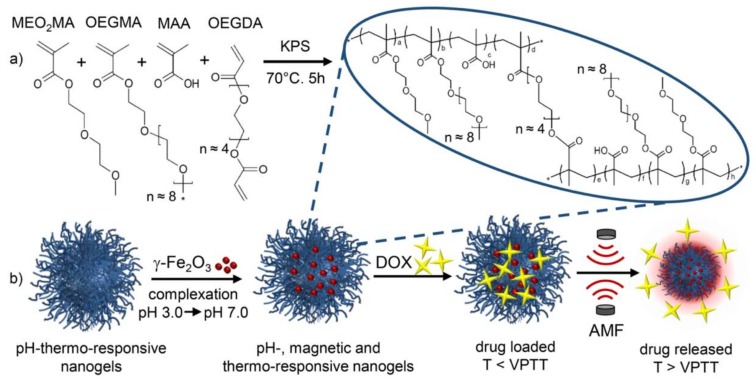
Schematic representation of thermo-responsive NGs loaded with maghemite. Reprinted with permission from ref. [155]. © 2017, American Chemical Society.

On the other hand, PHNGs can also be formed by performing a physical mixture between the organic network and the INPs suspension. This approach is especially useful when the polymeric network is a natural or commercial polymer. Zhou et al. [151], for example, coated maghemite NPs (Fe_2_O_3_NPs) with chitosan performing the mixture of both phases under ultrasonication. Recently, a hybrid NG system based on gold nanorods coated with polypeptides was developed for targeted drug delivery and tumor chemo-photothermal therapy [152]. A triblock-engineered polypeptide was immobilized on the surface of gold nanorods by electrostatic adsorption. Likewise, Boularas and his research team [153,154] demonstrated the successful formation of microgels with oligoethylenglycol methacrylate (OEG) monomers together with the physical incorporation of Fe_2_O_3_NPs. They were synthesized by conventional precipitation-polymerization and their sizes were approximately 500 nm. The nanogels showed a thermosensitive behavior and stability over time up to 100 h. Also, Cazares-Cortes et al. [155] reported pH-thermo-responsive OEG-based nanogels loaded with Fe_2_O_3_NPs. The synthetic route is shown in Figure 13. These nanogels presented a high drug loading efficiency of DOX and the release could be triggered by different stimulus such as pH and alternative magnetic field (AMF). Furthermore, they demonstrated the enhanced internalization of DOX in cancer cells and reduced cell viability by applying an AMF in the cells containing the NGs.

**Figure 14:**
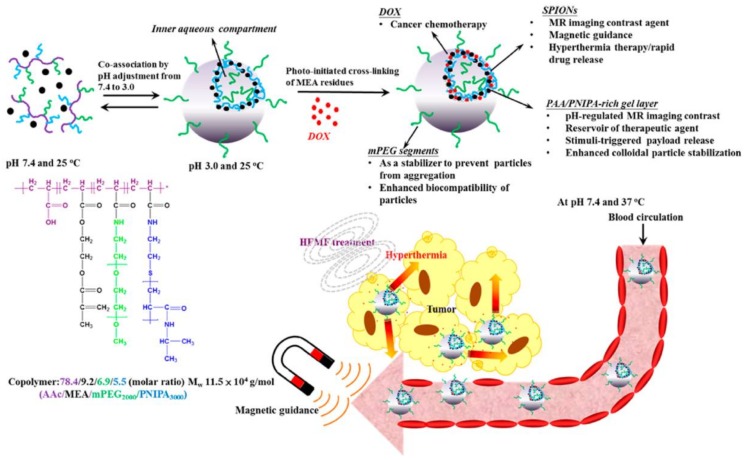
Development of the doxorubicin (DOX)-loaded hollow hybrid NGs serving as a multifunctional anticancer theranostic platform. Reprinted with permission from ref. [156].

Superparamagnetic hollow hybrid NGs were prepared by a physical mixture between polymer and magnetic nanoparticles to be tested as devices for stimuli-mediated MRI and cancer therapeutics [156] (Figure 14). These hybrid assemblies were prepared first by co-assembling citric acid-coated MNPs with the graft copolymer comprising AA and 2-methacryloylethyl acrylate (MEA) units as the backbone, and PEG and PNIPAM as the grafts in the aqueous phase of pH 3.0 in the hybrid vesicle structure. This was followed by in situ covalent stabilization through the photoinitiated polymerization of MEA residues to form ester crosslinks. The resultant hollow hybrid NGs exhibited such advantageous features as stimuli-mediated MRI contrast, hyperthermia, controlled drug delivery and cellular uptake, showing in vitro cytotoxic effect against tumor cells.

Alternatively, to perform the synthesis of the polymeric matrix and the INPs in situ, nanoparticle precursors can be loaded into the gel during polymerization. For example, Rajar et al. [157] reported the in situ synthesis of Fe_3_O_4_ nanorods with PNIPAM nanogels. Briefly, Fe_3_O_4_ precursors were added to the hydrogel-forming monomer solution before free radical polymerization was initiated. After gelation was completed, the composite was centrifuged and washed. To finally obtain Fe_3_O_4_ nanorods, an alkaline solution was added to the polymer suspension to obtain Fe_3_O_4_ nanorods by the co-precipitation method.

### 3.2. Covalent Hybrid Nanogels

So far, there are few reports of the covalent incorporation of INPs to the polymer matrix. One particularly interesting strategy in the development of CHNGs is the “grafting-through” approach. In this case, units of a polymerizable monomer are tethered to the surface of the INPs. Then, the covalently attached monomer is co-polymerized with other monomers present in the contacting solution yielding NGs, for example, by free radical polymerization. Here, the INP functionalized with monomers acts as a crosslinking agent. Free radical polymerization is also called uncontrolled radical polymerization and is one of the most commonly used techniques for obtaining NGs. This methodology employs an initiator and vinyl monomers. For SiO_2_ and Fe_3_O_4_ NPs, the functionalization of their surface with crosslinking moieties can be achieved by tethering a silane agent with vinyl terminals as MPS [140,158], vinyltrimethoxysilane [159], etc.

The grafting-through method leads to a homogeneous distribution of INPs inside the polymeric matrix. As an example of this, Schoth et al. [160] functionalized the surface of SiO_2_NPs either with MPS or octadecyl trimethoxysilane (ODTMS), and then carried out free radical miniemulsion polymerization using methyl methacrylate and butyl methacrylate as monomers. As a result, MPS-silica particles were homogeneously dispersed inside the polymer matrix whereas ODTMS-silica particles show a higher tendency to form aggregates in the polymer phase. This behavior can be explained by the chemical differences between the two functionalization agents: MPS is a polymerizable group able to copolymerize with free monomers and, therefore, form covalent bonds between the silica surface and the surrounding polymer matrix while ODTMS carries an inert alkyl chain. Hence, the covalent linkage between silica and polymer helps to suppress aggregation and leads to a homogeneous distribution of the SiO_2_NPs inside the matrix. A similar study was performed by functionalizing Fe_3_O_4_NPs with MPS, ODTMS, and oleic acid (OA), and performing polymerization with methyl methacrylate, styrene, and a mixture of styrene and 4-vinylpyridine [161]. Transmission and scanning electron micrographs show that MPS-Fe_3_O_4_NPs were distributed homogeneously within the polymers, while ODTMS and oleic acid lead to the formation of Janus particles.

Chen and colleagues modified the Fe_3_O_4_NPs with a silane agent with vinyl terminals to use it as a crosslinker of NGs of PNIPAM, which were obtained through the conventional thermoprecipitation methodology [140]. The sizes of the nanogels varied between ca. 1000 nm at 15 °C to 100–500 nm at 55 °C. These systems presented a thermosensitive behavior with a lower critical solution temperature (LCST) around 35 °C, this temperature being lower than the temperature at which the cells of a diseased tissue are found which is around 42 °C.

**Figure 15:**
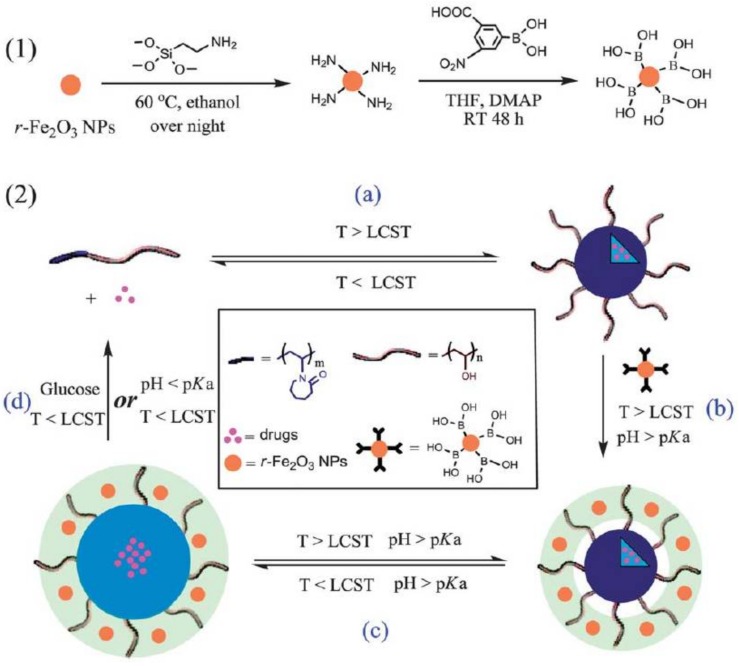
(**1**) Surface functionalization of the Fe_2_O_3_NPs with silane agents and boronic acid derivatives. (**2**) thermally-induced micelle formation and hydrophobic drug loading (**a**); crosslinking of the poly(vinyl alcohol) with the functionalized Fe_2_O_3_NPs (**b**); formation of drug-loaded NGs after cooling down below the LCST (**c**) and glucose-pH-triggered release of the drug molecules (**d**). Reprinted with permission from ref. [144]. © 2014, Royal Society of Chemistry.

Liu et al. [144] developed reversible crosslinked NGs based on poly((vinyl alcohol)-*b*-(*N*-vinylcaprolactam)) copolymers and Fe_2_O_3_NPs modified with boronic acid (Figure 15). In this case, the crosslinking was formed via boronate/diol bonding between boronic acid located onto the Fe_2_O_3_NPs and poly(vinyl alcohol) of the polymer matrix. Different hydrophobic drugs could be loaded in the nanodevice and released by different triggers like pH or glucose. In addition, due to the superparamagnetic nature of the maghemite, AMF application accelerate the drug release and the potential application of these systems as MRI contrast was evidenced.

“Click” chemistry, especially the azide-alkyne cycloaddition, has been widely used for the synthesis of NGs since it allows well-defined molecular structures and surprisingly improved mechanical properties, due to the specificity and the quantitative yields obtained [162]. Recently, Soleimani et al. synthetized graphene oxide NGs by using polysaccharide azides for cycloaddition reaction between cellulose azide and graphene oxide double bonds [163]. Additionally, our group in collaboration with Calderón’s group designed the synthesis of magnetic NGs through a methodology based on ultrasonication and orthogonal strain-promoted azide-alkine cycloaddition. For this purpose, we chose as building blocks magnetic nanoparticles decorated with bicyclononyne and thermosensitive diazide linear polyglycerol (N_3_-tPG-N_3_). Promising results were obtained, demonstrating the versatility of this synthetic tool. The magnetic properties of the system were analyzed, obtaining relaxation values like those of commercial MRI contrast agents. In addition, the system exhibited thermosensitive behavior. To evaluate the use of these NGs as capture devices for circulating metastasis cells (CTCs), we incorporated transferrin as targeting moieties. To this effect, the glycoprotein was modified with an azide PEG linker and incorporated into the matrix. The capture efficiencies of CTCs of all systems were evaluated, finding a dependence on both the length of the linker used, its relation to transferrin and the length of the polymer used. The optimal system was found for capturing CTCs with a capture efficiency of 80% using 5 kDa N_3_-tPG-N_3_ and the transferrin conjugate with the linker with chain length of 8 PEG units and a transferrin: linker ratio 1:3 [164,165].

Another approach for the synthesis of CHNGs consists of covalent linking between groups present in the polymer matrix and groups anchored onto the surface of INPs. In this sense, carboxymethyl chitosan was attached to MNPs by amidation reaction (CMCS-capped-MNPs) and then, CMCS-capped-MNPs were positively charged by adjusting the pH below 5.9 for its later intercalation within the montmorillonite layers by electrostatic interaction (CMCS-capped-MNP/MMT) [166]. DOX was effectively loaded into CMCS-capped-MNP/MMT giving a higher release rate at pH 5.0 than at pH 7.4, revealing that this drug delivery system had lower toxicity towards normal tissues. In an alternating magnetic gradient, CMCS-capped-MNP/MMT delivered appropriate amounts of the antitumor drug specifically to the cancer site because of the hyperthermia effect.

Using the same synthetic approach, hybrid NG formed by PAA and MSNPs were prepared performing the amidation between the amino groups of MSNP and the carboxyl groups of PAA. These hybrid NGs were tested as a pH-responsive controlled drug delivery system using DOX as a model drug to assess the drug loading and releasing behaviors. High drug loading efficiency was observed, due to the strong electrostatic interaction between PAA and DOX. The release rate of DOX was pH dependent, increasing with the decrease of pH [167].

Carboxyl groups functionalized MSNPs can also be achieved, for example, by performing the reaction between amino-functionalized nanoparticles and succinic acid. Particularly, this surface modification facilitated the covalent linkage to branched polyethylenimine. Recently, Sun et al. used this approach for the controlled synthesis of a MSNPs/organosilica nanosystem, in which large and small molecules (siRNA and DOX, respectively) were separately encapsulated in large and small mesopores. This design allowed sequential release of both therapeutic agents [168].

## 4. Core-Shell Nanoparticles

Core-shell nanoparticles can be achieved by functionalizing of INPs with organic molecules such as dendrimers or dendrons [169,170,171], enzymes [172], antitumor drugs [173], and disulfide silanes [174], etc. However, here we will only refer to those systems in which the shell is made of polymer.

It is worth noticing that some authors refer to core-brush nanoparticles with high density of tethered chains polymer as a core-brush shell or simply core-shell nanostructures. Once more, the properties of these NPs will be defined by the chemical structure and the thickness of the organic layer, as well as by the nature of the core, as previously stated. Core-brush shell nanoparticles can be achieved by the above described synthetic routes, grafting-from and to approaches, which assure the presence of only one inorganic core per individual nanoparticle. Except for the previous case, we have not found in the literature general synthetic routes that lead to the formation of a core-shell architecture guaranteeing only one inorganic core. Instead, core-shell (not core-brush shell) structures can be produced by carefully handling the experimental conditions of the polymerization process as we will see in the examples below. For example, for the synthesis of Au@PNIPAM thermos-sensitive nanostructures the polymer coating on gold cores is achieved by using cationic surfactant (cetyltrimethylammonium bromide) bilayer as a strategy [175]. This bilayer provides a relatively thick hydrophobic environment in which the physical adsorption of water-insoluble styrene and divinylbenzene can take place. The addition of a suitable initiator leads to the polymerization of styrene and divinylbenzene around the gold nanoparticles while maintaining non-polymerized vinyl groups available at the surface. Then, NIPAM polymerization on the polystyrene-coated nanoparticles can be performed using a standard surfactant-free polymerization process. Transmission electron microscopy images clearly showed isolated core-shell nanostructures obtained by this protocol.

The grafting-through approach can be also used for the synthesis of core-shell hybrid nanogels. In this case, to achieve the core-shell architecture, the polymerization between vinyl-cores and free monomers must be favored over polymerization between cores. Following this approach, PNIPAM-grafted MSNPs were synthetized by the co-polymerization of NIPAM and vinyl-functionalized MSNPs using free radical polymerization by the ultrasound-induced mini emulsion technique. The average thickness of the polymer coating was in the order of 90 nm. The synthesized hybrid material exhibited superior pH sensitivity, biocompatibility and antibacterial activity compared to the materials prepared by other techniques [158]. Another example is the synthesis of core-shell hybrid nanogels formed by a nanostructured inorganic silica core and an organic pH-responsive PDEAEMA coating via free radical co-polymerization through an oil-in-water emulsion technique of the cationic monomer 2-(diethylamino)ethyl methacrylate (DEAEM) and the monomeric silica precursor vinyltrimethoxysilane in the presence of PEGMA and triethylene glycol dimethacrylate linkers. A fluorescent dye (fluorescein isothiocyanate) was incorporated within the silica lattice during the synthesis procedure. Therefore, this pH-responsive fluorescent nanogel was tested as a non-viral carrier for siRNA therapeutics. Since the tertiary amine group of the PDEAEMA shell is protonated in an acidic environment, the hydrodynamic volume of the nanogel increased when the pH changed from 7 to 5. SiRNA release was accelerated in acidic conditions despite the higher affinity of the hybrid nanogel and the siRNA at these conditions, likely because of hydrogel swelling, which increased the solubility of the coating and favored the diffusion of the payload into the surrounding environment. Finally, CXCR4 siRNA was efficiently delivered into the cytoplasm of MDA-MB-231 breast cancer cells and inhibited the protein expression of CXCR4 with an efficacy comparable to that of a commercial transfection reagent. Moreover, the hybrid nanoparticles had an excellent efficacy in delivering siRNA in vivo because the intravenous administration of siRNA-loaded nanoparticles demonstrated a preferential accumulation at the tumor site, which resulted in a reduction of CXCR4 expression [159].

Another example is the synthesis of core-shell hybrid nanogels formed by SiO_2_NPs and redox-responsive polymers. For instance, Qiao et al. synthetized a hybrid nanogel, which was tested as a highly efficient theranostic agent based on MSNPs coated with a redox responsive polymer. Here, the vinyl terminals were anchored to MSNPs in steps. First the MSNPs were modified with an amino group via silane coupling agent of APTES. Next the vinyl groups were obtained by the reaction of *N*-acryloyloxy succinimide with MSNP-NH_2_. Finally, a redox stimuli-responsive hydrogel coating was obtained via laccase-mediated polymerization of *N*,*N*-dimethylacrylamide as the gel network and *N*,*N*-bis(acryloyl)cystamine with disulfides (S-S) as the responsive crosslinker. The laccase system can benefit the surface polymerization and, consequently, the shell formation (without crosslinking), due to the slow generation of free radicals. The hydrophilic hydrogel networks in the outer layer and abundant mesoporous channels of MSNPs were simultaneously loaded with the hydrophilic antitumor drug DOX and hydrophobic ultrasound (US) synergistic agent perfluorohexane (PFH). Therefore, this NG could responsively release DOX and enhance US imaging after US-triggered, temperature-sensitive PFH acoustic droplet vaporation when exposed to a reducing environment, for example, in tumors [176].

**Figure 16:**
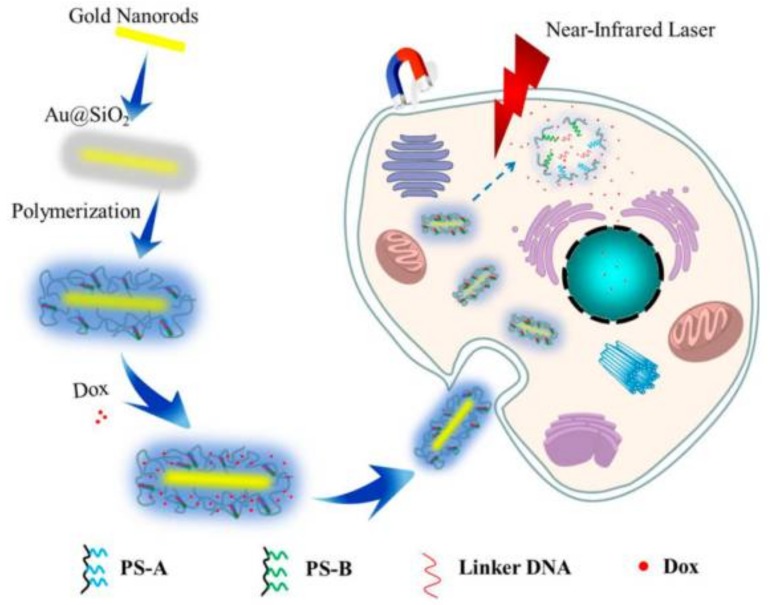
Schematic illustration of magnetic core−shell DNA microgels. Reprinted with permission from ref. [177]. © 2014, Royal Society of Chemistry.

Wang et al. synthetized dual-responsive core-shell nanogels that were composed of a core of Au nanorods and a shell of magnetic ionic liquid and DNA moieties in the crosslinking network simultaneously, as effective drug delivery vectors (Figure 16) [177]. First, Au nanorods were coated with a silica shell to prevent its aggregation. Secondly, a silicane-coupling agent (MPS) was used to endow the surface of the silica shell with abundant C=C bonds. Finally, the growth of linear polymeric chains was initiated through photoinitiated free-radical polymerization with the magnetic ionic liquids monomer (3-*N*-butyl-1-vinylimidazolium trichloromonobromoferrate) and acrydite-modified DNA, which not only acted as crosslinker agent but could also serve as a gatekeeper to regulate the release of drug. The obtained core-shell nanoparticles were tested for magneto-manipulated cancer therapy. Doxorubicin hydrochloride (DOXh) was used as a drug for controlled release tests. An internalization study and MTT assay demonstrated that the DOXh-loaded core−shell DNA microgels could efficiently uptake into cancer cells and enhance the cytotoxicity of cancer cells controlled by near-IR laser.

## 5. Concluding Remarks and Future Perspectives

The preparation of decorated surfaces of NP with molecules or polymers with simple structure might not be enough for their efficient application in nanomedicine. In the Section 2, Section 3 and Section 4, we have shown some examples of controlled synthetic approaches to produce hybrid nanomaterials with interesting potential in biomedical applications. Therefore, for the design and development of hybrid nanomaterials capable of displaying relevant functions in the biomedical field, it is important to comprehensively control the composition of the material and their architecture and carefully examine the structure–property relationships.

Three factors are the most important when choosing a system as nanocarrier or nanodevice in medical applications: size, stability, and functionality (functional groups and charge).

Particle size is a crucial factor for bio-applications. Since particle size is intrinsically related to the rate of clearance from the blood circulation, it is important for controlling tumor accumulation kinetics and for preventing diffusion back into the systemic vascular bed [1]. For instance, smaller particles (in the range of 50–300 nm) have slower removal from the circulation than larger ones [1]. From the comparative analysis of bibliographic results, we have not found a clear tendency between the synthetic methodology (grafting-from, to or through) and the particle size. Size is the consequence of many variables like the nature of monomers, crosslinking degree, polymerization technique, characteristics of the core, among others. Nonetheless, covalent polymer coating confers more stability to the final structure than those obtained by physical interactions because the covalent anchoring of inorganic NPs to the polymer chains reduces the possibility of desorption under in vivo conditions.

The surface functionality of the NPs is very dependent on the type of monomers chosen. The functional groups in the organic component of the nanocarriers will be responsible for the charge, smart behavior, and act as sites for the anchoring of target molecules and/or drug to delivery. Another relevant factor to keep in mind is that the polymerization strategy like grafting-to, from or through protocols might result in different superficial densities of functional groups. Grafting density plays an important role in establishing the regime in which the macromolecular system operates [53], which is therefore closely related to their smart behavior in response to stimuli.

Thermo-responsive brushes are attractive building blocks for design and fabrication of smart nanocarriers for controlling drug delivery and theranosis. We will focus on these systems to show that the brush architecture can influence the smart behavior. “Grafting-from” techniques usually yield higher grafted layer densities compared to “grafting-to” [36]. Therefore, for the low grafting densities typically obtained by “grafting-to”, core-core interactions dominate above the LCST which then lead to aggregation. For the higher grafting densities achieved by “grafting-from”, the brushes keep mobile, soluble chain ends even above the LCST, which can reduce or prevent aggregation [102]. Specifically in these systems, previous publications have demonstrated the difficulty of some core-brush systems to identify the LCST temperatures and smart thermo behaviors [102]. One of the problems derived of brush architecture is the reduced chain motilities from covalent immobilization. Therefore, the interchain interactions make the collapse of the grafted polymers weaker than that of a free polymer chain in solution. The broader thermal transitions of polymer brushes on the nanoparticles might be also related to the different degrees of freedom of segments along the grafted polymer chains [163]. Immobilization of the initiator on the surface of NPs is a key factor in the grafting-from approach. The attaching of an initiator to a surface leads to lower concentrations than in solution [164]. Consequently, brush density depends on the density of the initiation site [165].

On the other hand, hybrid nanogels are interesting materials for biomedical applications due to their unique properties, such as high encapsulation efficiency due to their porous structure and protection of active agents from degradation, which make them ideal candidates as drug delivery or theranostic systems [178].

In this work, we have reviewed the recent progress in the design and synthetic approaches on NPs emphasizing those procedures that lead to each type of architecture, be it core-brush, hybrid nanogels and core-shell. In all cases, we have shown recent examples of the use of these hybrid nanomaterials in nanomedicine. Drug delivery nanocarriers can lead to enhanced tumor targeting efficiency, improved drug solubility, reduced side effects, increased drug half-life in the body, and therefore to a more efficient cancer therapy [179]. Another important field in nanomedicine is the detection and diagnosis of diseases at early stages. This can be achieved by the molecular imaging of cells and tissues by MRI, computer tomography, nuclear imaging and single photon emission-computed tomography, optical fluorescent imaging, and ultrasound [25]. Finally, theranostics combine therapeutic and diagnostic capabilities into a single platform to enable more specific and individualized therapies for various diseases and damages [25].

**Figure 17:**
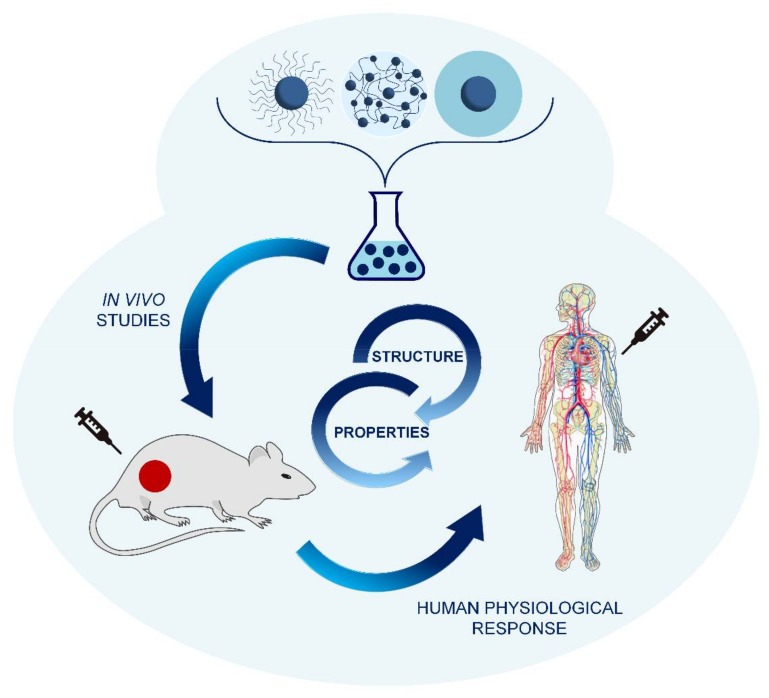
Schematic illustration to highlight the importance of understanding the structure/property relationship of the different systems to achieve successful in vitro and in vivo applications.

Updated discussions of the advantages and disadvantages of the different synthetic methodologies published up to now are useful for a better understanding of how the structure/property relationship of the different systems can be handled to achieve successful in vitro and in vivo applications. Nevertheless, more work is currently needed for achieving a full use of hybrid engineered nanomaterials in biomedical applications. Overall, we consider that core-brush nanomaterials obtained by grafting-from approach are promising materials because this way it is possible to control the material morphology. In this sense, SI-ATRP is useful for the synthesis of hybrid core-brush nanostructures since it leads to controlled molecular weight, low polydispersity, and varied functionality. However, the use of metal catalysts, such as copper complexes, is a limitation for biomedical applications [180]. In this context, we strongly believe that metal-free ATRP is a potential technique for the development of core-brush systems for the future. Our observation is supported by some recent original reports that show improvements in this field, particularly in electrochemically mediated ATRP [40,180], photoinduced metal-free ATRP [181], and enzyme mimetic catalytic ATRP [182]. In addition, covalent hybrid nanogels are also promising materials for applications in nanomedicine. Grafting-through approach via free radical polymerization is a robust technique that involves simple and inexpensive procedures leading to high yields. However, there are still many significant challenges researchers need to address in order to improve the suitability of these materials for clinical application. In this context, Figure 17 illustrates our wishes towards future developments in this area. We expect that this review will inspire further studies in the design of smart, responsive hybrid nanodevices for drug delivery, specifically therapies of high efficacy and diagnosis.

## Figures and Tables

**Table 1:** Most used halogen-ended silanization agents for SI-ATRP.

Silanization Agent	Ref.
3-(dimethylethoxysilyl)propyl-2-bromoisobutyrate	[61]
[11-(2-bromo-2-methyl)-propionyloxy]undecyltrichlorosilane	[62,63]
2-bromo-2-methyl-*N*-(3-(triethoxysilyl)propyl)propanamide	[64]
[3-(2-bromo-2-methyl)propionyloxylpropyl]trimethoxysilane	[65]
[(2-bromo-2-methyl)-propionyloxyhexyl]triethoxysilane	[66]
[(p-chloromethyl)phenyl]trimethoxy-silane	[67]

## References

[1] Bae Y.H., Park K. (2011). Targeted drug delivery to tumors: Myths, reality and possibility. J. Control. Release.

[2] Oshiro Junior J., Abuçafy M.P., Manaia E.B., da Silva B.L., Chiari-Andréo B., Chiavacci L.A. (2016). Drug Delivery Systems Obtained from Silica Based Organic-Inorganic Hybrids. Polymers.

[3] Bregoli L., Movia D., Gavigan-imedio J.D., Lysaght J., Reynolds J., Prina-mello A. (2016). Nanomedicine applied to translational oncology: A future perspective on cancer treatment. Nanomed. Nanotechnol. Biol. Med..

[4] Komiyama M., Yoshimoto K., Sisido M., Ariga K. (2017). Chemistry can make strict and fuzzy controls for bio-systems: DNA nanoarchitectonics and cell-macromolecular nanoarchitectonics. Bull. Chem. Soc. Jpn..

[5] Ariga K., Li J., Fei J., Ji Q., Hill J.P. (2016). Nanoarchitectonics for Dynamic Functional Materials from Atomic-/Molecular-Level Manipulation to Macroscopic Action. Adv. Mater..

[6] Ariga K., Minami K., Ebara M., Nakanishi J. (2016). What are the emerging concepts and challenges in NANO Nanoarchitectonics, hand-operating nanotechnology and mechanobiology. Polym. J..

[7] Ariga K., Ji Q., Nakanishi W., Hill J.P., Aono M. (2015). Nanoarchitectonics: A new materials horizon for nanotechnology. Mater. Horiz..

[8] Aono M., Ariga K. (2016). The Way to Nanoarchitectonics and the Way of Nanoarchitectonics. Adv. Mater..

[9] Mazur J., Roy K., Kanwar J.R. (2018). Recent advances in nanomedicine and survivin targeting in brain cancers. Nanomedicine.

[10] Lutz J.F., Lehn J.M., Meijer E.W., Matyjaszewski K. (2016). From precision polymers to complex materials and systems. Nat. Rev. Mater..

[11] Matyjaszewski K., Tsarevsky N.V. (2014). Macromolecular engineering by atom transfer radical polymerization. J. Am. Chem. Soc..

[12] Zhao Z., Lou S., Hu Y., Zhu J., Zhang C. (2017). A Nano-in-Nano Polymer-Dendrimer Nanoparticle-Based Nanosystem for Controlled Multidrug Delivery. Mol. Pharm..

[13] Sahle F.F., Giulbudagian M., Bergueiro J., Lademann J., Calderón M. (2017). Dendritic polyglycerol and *N*-isopropylacrylamide based thermoresponsive nanogels as smart carriers for controlled delivery of drugs through the hair follicle. Nanoscale.

[14] Giulbudagian M., Hönzke S., Bergueiro J., Isik D., Schumacher F., Saeidpour S., Lohan S.B., Meinke M.C., Teutloff C., Schaefer-Korting M. (2017). Enhanced Topical Delivery of Dexamethasone by beta-Cyclodextrin Decorated Thermoresponsive Nanogels. Nanoscale.

[15] Zhao Z., Harris B., Hu Y., Harmon T., Pentel P.R., Ehrich M., Zhang C. (2018). Rational incorporation of molecular adjuvants into a hybrid nanoparticle-based nicotine vaccine for immunotherapy against nicotine addiction. Biomaterials.

[16] Zhao Z., Powers K., Hu Y., Raleigh M., Pentel P., Zhang C. (2017). Engineering of a hybrid nanoparticle-based nicotine nanovaccine as a next-generation immunotherapeutic strategy against nicotine addiction: A focus on hapten density. Biomaterials.

[17] Zhu J., Niu Y., Li Y., Gong Y., Shi H., Liu Y., Xu Q., Huo Q. (2017). Stimuli-Responsive delivery Vehicles Based on Mesoporous Silica Nanoparticles: Recent Advances and Challenges. J. Mater. Chem. B.

[18] Ulbrich K., Holá K., Šubr V., Bakandritsos A., Tuček J., Zbořil R. (2016). Targeted Drug Delivery with Polymers and Magnetic Nanoparticles: Covalent and Noncovalent Approaches, Release Control, and Clinical Studies. Chem. Rev..

[19] Kwizera E.A., Chaffin E., Wang Y., Huang X. (2017). Synthesis and properties of magnetic-optical core–shell nanoparticles. RSC Adv..

[20] Adeli M., Soleyman R., Beiranvand Z., Madani F. (2013). Carbon nanotubes in cancer therapy: A more precise look at the role of carbon nanotube–polymer interactions. Chem. Soc. Rev..

[21] Momper R., Steinbrecher J., Dorn M., Rörich I., Bretschneider S., Tonigold M., Ramanan C., Ritz S., Mailänder V., Landfester K. (2018). Enhanced photoluminescence properties of a carbon dot system through surface interaction with polymeric nanoparticles. J. Colloid Interface Sci..

[22] Donskyi I., Drüke M., Silberreis K., Lauster D., Ludwig K., Kühne C., Unger W., Böttcher C., Herrmann A., Dernedde J. (2018). Interactions of Fullerene-Polyglycerol Sulfates at Viral and Cellular Interfaces. Small.

[23] Baek S., Singh R.K., Khanal D., Patel K.D., Lee E.-J., Leong K.W., Chrzanowski W., Kim H.-W. (2015). Smart multifunctional drug delivery towards anticancer therapy harmonized in mesoporous nanoparticles. Nanoscale.

[24] Lee Y.K., Choi E.J., Webster T.J., Kim S.H., Khang D. (2014). Effect of the protein corona on nanoparticles for modulating cytotoxicity and immunotoxicity. Int. J. Nanomed..

[25] Singh R.K., Patel K.D., Leong K.W., Kim H. (2017). Progress in Nanotheranostics Based on Mesoporous Silica Nanomaterial Platforms. Appl. Mater. Interfaces.

[26] Bobo D., Robinson K.J., Islam J., Thurecht K.J., Corrie S.R. (2016). Nanoparticle-Based Medicines: A Review of FDA-Approved Materials and Clinical Trials to Date. Pharm. Res..

[27] Kunzmann A., Andersson B., Thurnherr T., Krug H., Scheynius A., Fadeel B. (2011). Toxicology of engineered nanomaterials: Focus on biocompatibility, biodistribution and biodegradation. Biochim. Biophys. Acta.

[28] Shen Z., Nieh M.-P., Li Y. (2016). Decorating Nanoparticle Surface for Targeted Drug Delivery: Opportunities and Challenges. Polymers.

[29] Zhang Z., Zhang P., Wang Y., Zhang W. (2016). Recent advances in organic–inorganic well-defined hybrid polymers using controlled living radical polymerization techniques. Polym. Chem..

[30] Schejtman S.D.G., Brunetti V., Martinelli M., Strumia M.C. (2017). Chemistry of hybrid multifunctional and multibranched composites. Hybrid Polymer Composite Materials.

[31] Riehemann K., Schneider S.W., Luger T.A., Godin B., Ferrari M., Fuchs H. (2009). Nanomedicine—Challenge and perspectives. Angew. Chem. Int. Ed..

[32] Farokhzad R., Langer O.C. (2009). Impact of Nanotechnology on Drug Delivery. ACS Nano.

[33] Latorre A., Couleaud P., Aires A., Cortajarena A.L., Somoza Á. (2014). Multifunctionalization of magnetic nanoparticles for controlled drug release: A general approach. Eur. J. Med. Chem..

[34] Lim W.Q., Phua S.Z.F., Xu H.V., Sreejith S., Zhao Y. (2016). Recent advances in multifunctional silica-based hybrid nanocarriers for bioimaging and cancer therapy. Nanoscale.

[35] Bressler E.M., Kim J., Shmueli R.B., Mirando A.C., Bazzazi H., Lee E., Popel A.S., Pandey N.B., Green J.J. (2018). Biomimetic peptide display from a polymeric nanoparticle surface for targeting and antitumor activity to human triple-negative breast cancer cells. J. Biomed. Mater. Res. Part A.

[36] Conzatti G., Cavalie S., Combes C., Torrisani J., Carrere N., Tourrette A. (2017). Biointerfaces PNIPAM grafted surfaces through ATRP and RAFT polymerization: Chemistry and bioadhesion. Colloids Surf. B Biointerfaces.

[37] Wu L., Glebe U., Böker A. (2015). Surface-initiated controlled radical polymerizations from silica nanoparticles, gold nanocrystals, and bionanoparticles. Polym. Chem..

[38] Nitschke M. (2008). Polymer Surfaces and Interfaces.

[39] Garcia I., Zafeiropoulos N.E., Janke A., Tercjak A., Eceiza A., Stamm M., Mondragon I. (2007). Functionalization of Iron Oxide Magnetic Nanoparticles with Poly(methyl methacrylate) Brushes Via Grafting-From Atom Transfer Radical Polymerization. J. Polym. Sci. Part A Polym. Chem..

[40] Yang D.-P., Oo M.N.N.L., Deen G.R., Li Z., Loh X.J. (2017). Nano-Star-Shaped Polymers for Drug Delivery Applications. Macromol. Rapid Commun..

[41] Zetterlund P.B., Thickett S.C., Perrier S., Bourgeat-Lami E., Lansalot M. (2015). Controlled/Living Radical Polymerization in Dispersed Systems: An Update. Chem. Rev..

[42] De Gennes P.G. (1987). Polymers at an interface; a simplified view. Adv. Colloid Interface Sci..

[43] Zoppe J.O., Ataman N.C., Mocny P., Wang J., Moraes J., Klok H. (2017). Surface-Initiated Controlled Radical Polymerization: State-of-the-Art, Opportunities, and Challenges in Surface and Interface Engineering with Polymer Brushes. Chem. Rev..

[44] Chmielarz P., Yan J., Krys P., Wang Y., Wang Z., Bockstaller M.R., Matyjaszewski K. (2017). Synthesis of Nanoparticle Copolymer Brushes via Surface-Initiated seATRP. Macromolecules.

[45] Jenkins A.D., Jones R.G., Moad G. (2010). Terminology for reversible-deactivation radical polymerization previously called “controlled” radical or “living” radical polymerization ( IUPAC Recommendations 2010). Pure Appl. Chem..

[46] Jiang Y., Chen J., Deng C., Suuronen E.J., Zhong Z. (2014). Click hydrogels, microgels and nanogels: Emerging platforms for drug delivery and tissue engineering. Biomaterials.

[47] Li N., Binder W.H. (2011). Click-chemistry for nanoparticle-modification. J. Mater. Chem..

[48] Fournier D., Hoogenboom R., Schubert U.S. (2007). Clicking polymers: A straightforward approach to novel macromolecular architectures. Chem. Soc. Rev..

[49] Meldal M., Tomøe C.W. (2008). Cu-catalyzed azide—Alkyne cycloaddition. Chem. Rev..

[50] Koo H., Lee S., Na J.H., Kim S.H., Hahn S.K., Choi K., Kwon I.C., Jeong S.Y., Kim K. (2012). Bioorthogonal copper-free click chemistry in vivo for tumor-targeted delivery of nanoparticles. Angew. Chem. Int. Ed..

[51] Kolb H.C., Finn M.G., Sharpless K.B. (2001). Click Chemistry: Diverse Chemical Function from a Few Good Reactions. Angew. Chem. Int. Ed..

[52] Golas P.L., Matyjaszewski K. (2010). Marrying click chemistry with polymerization: Expanding the scope of polymeric materials. Chem. Soc. Rev..

[53] Giussi J.M., Cortez M.L., Marmisollé W.A., Azzaroni O. (2017). Functionalization of Surfaces Using Polymer Brushes: An Overview of Techniques, Strategies, and Approaches. Polymer and Biopolymer Brushes: For Materials Science and Biotechnology 2 Volume Set.

[54] Matyjaszewski K. (2012). Atom Transfer Radical Polymerization (ATRP): Current status and future perspectives. Macromolecules.

[55] He W., Jiang H., Zhang L., Cheng Z., Zhu X. (2013). Atom transfer radical polymerization of hydrophilic monomers and its applications. Polym. Chem..

[56] Matyjaszewski K., Xia J. (2001). Atom transfer radical polymerization. Chem. Rev..

[57] Siegwart D.J., Oh J.K., Matyjaszewski K. (2012). ATRP in the design of functional materials for biomedical applications. Prog. Polym. Sci..

[58] Boyer C., Corrigan N.A., Jung K., Nguyen D., Nguyen T.-K., Adnan N.N.M., Oliver S., Shanmugam S., Yeow J. (2015). Copper-Mediated Living Radical Polymerization (Atom Transfer Radical Polymerization and Copper(0) Mediated Polymerization): From Fundamentals to Bioapplications. Chem. Rev..

[59] Hui C.M., Pietrasik J., Schmitt M., Mahoney C., Choi J., Bockstaller M.R., Matyjaszewski K. (2014). Surface-initiated polymerization as an enabling tool for multifunctional (Nano-)engineered hybrid materials. Chem. Mater..

[60] Zhou Y., Wang S., Ding B., Yang Z. (2008). Modification of magnetite nanoparticles via surface-initiated atom transfer radical polymerization (ATRP). Chem. Eng. J..

[61] Perruchot C., Khan M.A., Kamitsi A., Armes S.P., von Werne T., Patten T.E. (2001). Synthesis of well-defined, polymer-grafted silica particles by aqueous ATRP. Langmuir.

[62] Czaun M., Hevesi L., Takafuji M., Ihara H. (2008). A novel approach to magneto-responsive polymeric gels assisted by iron nanoparticles as Nano cross-linkers. Chem. Commun..

[63] Li D., Jones G.L., Dunlap J.R., Hua F., Zhao B. (2006). Thermosensitive Hairy Hybrid Nanoparticles Synthesized by Surface-Initiated Atom Transfer Radical Polymerization. Langmuir.

[64] Sun Y., Ding X., Zheng Z., Cheng X., Hu X., Peng Y. (2007). Surface initiated ATRP in the synthesis of iron oxide/polystyrene core/shell nanoparticles. Eur. Polym. J..

[65] Wu L., Glebe U., Böker A. (2016). Synthesis of Hybrid Silica Nanoparticles Densely Grafted with Thermo and pH Dual-Responsive Brushes via Surface-Initiated ATRP. Macromolecules.

[66] Morinaga T., Honma S., Ishizuka T., Kamijo T., Sato T., Tsujii Y. (2016). Synthesis of Monodisperse Silica Particles Grafted with Concentrated Ionic Liquid-Type Polymer Brushes by Surface-Initiated Atom Transfer Radical Polymerization for Use as a Solid State. Polymers.

[67] Marten G.U., Gelbrich T., Schmidt A.M. (2010). Hybrid biofunctional nanostructures as stimuli-responsive catalytic systems. Beilstein J. Org. Chem..

[68] Lu X., Jiang R., Fan Q., Zhang L., Zhang H., Yang M., Ma Y., Wang L., Huang W. (2012). Fluorescent-magnetic poly(poly(ethyleneglycol)monomethacrylate)-grafted Fe_3_O_4_ nanoparticles from post-atom-transfer-radical-polymerization modification: Synthesis, characterization, cellular uptake and imaging. J. Mater. Chem..

[69] Liu J., He W., Zhang L., Zhang Z., Zhu J., Yuan L., Chen H., Cheng Z., Zhu X. (2011). Bifunctional nanoparticles with fluorescence and magnetism via surface-initiated AGET ATRP mediated by an iron catalyst. Langmuir.

[70] Jiang L., Bagán H., Kamra T., Zhoua T., Ye L. (2016). Nanohybrid polymer brushes on silica for bioseparation. J. Mater. Chem. B.

[71] Sun J.T., Hong C.Y., Pan C.Y. (2010). Fabrication of PDEAEMA-coated mesoporous silica nanoparticles and pH-responsive controlled release. J. Phys. Chem. C.

[72] Arica T.A., Ayas E., Arica M.Y. (2017). Magnetic MCM-41 silica particles grafted with poly(glycidylmethacrylate) brush: Modification and application for removal of direct dyes. Microporous Mesoporous Mater..

[73] Fan Q.-L., Neoh K.-G., Kang E.-T., Shuter B., Wang S.-C. (2007). Solvent-free atom transfer radical polymerization for the preparation of poly(poly(ethyleneglycol) monomethacrylate)-grafted Fe_3_O_4_ nanoparticles: Synthesis, characterization and cellular uptake. Biomaterials.

[74] Li G., Zeng D.L., Wang L., Zong B., Neoh K.G., Kang E.T. (2009). Hairy hybrid nanoparticles of magnetic core, fluorescent silica shell, and functional polymer brushes. Macromolecules.

[75] Iacono M., Heise A. (2015). Stable Poly(methacrylic acid) Brush Decorated Silica Nano-Particles by ARGET ATRP for Bioconjugation. Polymers.

[76] Huang L., Liu M., Mao L., Xu D., Wan Q., Zeng G., Shi Y., Wen Y., Zhang X., Wei Y. (2017). Applied Surface Science Preparation and controlled drug delivery applications of mesoporous silica polymer nanocomposites through the visible light induced. Appl. Surf. Sci..

[77] Yu E., Lo A., Jiang L., Petkus B., Ercan N.I., Stroeve P. (2017). Improved controlled release of protein from expanded-pore mesoporous silica nanoparticles modified with co-functionalized poly(*n*-isopropylacrylamide) and poly(ethylene glycol) (PNIPAM-PEG). Colloids Surf. B Biointerfaces.

[78] Bergueiro J., Calderon M. (2015). Thermoresponsive Nanodevices in Biomedical Applications. Macromol. Biosci..

[79] Isojima T., Lattuada M., Sande J.B.V., Hatton T.A. (2008). Reversible clustering of pH- and temperature-responsive Janus magnetic nanoparticles. ACS Nano..

[80] Yar Y., Khodadust R., Akkoc Y., Utkur M., Saritas E.U., Gozuacik D., Acar H.Y. (2018). Development of tailored SPION-PNIPAM nanoparticles by ATRP for dually responsive doxorubicin delivery and MR imaging. J. Mater. Chem. B.

[81] Huang C., Neoh K.G., Kang E.T. (2012). Combined ATRP and “click” chemistry for designing stable tumor-targeting superparamagnetic iron oxide nanoparticles. Langmuir.

[82] Zhang Y., Ang C.Y., Li M., Tan S.Y., Qu Q., Luo Z., Zhao Y. (2015). Polymer-Coated Hollow Mesoporous Silica Nanoparticles for Triple-Responsive Drug Delivery. ACS Appl. Mater. Interfaces.

[83] Moad G., Rizzardo E., Thang S.H. (2008). Radical addition-fragmentation chemistry in polymer synthesis. Polymer.

[84] Hill M.R., Carmean R.N., Sumerlin B.S. (2015). Expanding the Scope of RAFT Polymerization: Recent Advances and New Horizons. Macromolecules.

[85] Perrier S. (2017). 50th Anniversary Perspective: RAFT Polymerization—A User Guide. Macromolecules.

[86] Zhao Y., Perrier S. (2007). Reversible addition-fragmentation chain transfer graft polymerization mediated by fumed silica supported chain transfer agents. Macromolecules.

[87] Liu J., Zhang L., Shi S., Chen S., Zhou N., Zhang Z., Cheng Z., Zhu X. (2010). A novel and universal route to SiO2-supported organic/inorganic hybrid noble metal nanomaterials via surface raft polymerization. Langmuir.

[88] Li Q., Zhang L., Bai L., Zhang Z., Zhu J., Zhou N., Cheng Z., Zhu X. (2011). Multistimuli-responsive hybrid nanoparticles with magnetic core and thermoresponsive fluorescence-labeled shell via surface-initiated RAFT polymerization. Soft Matter.

[89] Qu Z., Hu F., Chen K., Duan Z., Gu H., Xu H. (2013). A facile route to the synthesis of spherical poly(acrylic acid) brushes via RAFT polymerization for high-capacity protein immobilization. J. Colloid Interface Sci..

[90] Moraes J., Ohno K., Maschmeyer T., Perrier S. (2013). Synthesis of silica–polymer core–shell nanoparticles by reversible addition–fragmentation chain transfer polymerization. Chem. Commun..

[91] Rotzoll R., Nguyen D.H., Vana P.I. (2009). Controlled radical polimerization trithiocarbonates containing trimethoxysilyl functionalities as mediating agents in reversible addition-fragmentation chain transfer (RAFT) polymerization of methyl acrylate. Macromol. Symp..

[92] Rotzoll R., Philipp V. (2008). Synthesis of Poly(methyl acrylate) Loops Grafted onto Silica Nanoparticles via Reversible Addition-Fragmentation Chain Transfer Polymerization. J. Polym. Sci. Part A Polym. Chem..

[93] Ohno K., Ma Y., Huang Y., Mori C., Yahata Y., Tsujii Y., Maschmeyer T., Moraes J., Perrier S. (2011). Surface-initiated reversible addition-fragmentation chain transfer (RAFT) polymerization from fine particles functionalized with trithiocarbonates. Macromolecules.

[94] Li C., Benicewicz B.C. (2005). Synthesis of well-defined polymer brushes grafted onto silica nanoparticles via surface reversible addition-fragmentation chain transfer polymerization. Macromolecules.

[95] Ranjan R., Brittain W.J. (2008). Synthesis of high density polymer brushes on nanoparticles by combined RAFT polymerization and click chemistry. Macromol. Rapid Commun..

[96] Jiao Y., Akcora P. (2014). Accelerated brush growth on nanoparticle surfaces by reversible addition-fragmentation chain transfer polymerization. J. Polym. Sci. Part A Polym. Chem..

[97] Moraes J., Ohno K., Maschmeyer T., Perrier S. (2013). Monodisperse, charge-stabilized, core-shell particles via silica-supported reversible addition-fragmentation chain transfer polymerization for cell imaging. Chem. Mater..

[98] Neugebauer D. (2015). Two decades of molecular brushes by ATRP. Polymer.

[99] Keddie D.J. (2014). A guide to the synthesis of block copolymers using reversible-addition fragmentation chain transfer (RAFT) polymerization. Chem. Soc. Rev..

[100] Tardy A., Nicolas J., Gigmes D., Lefay C., Guillaneuf Y. (2017). Radical Ring-Opening Polymerization: Scope, Limitations, and Application to (Bio)Degradable Materials. Chem. Rev..

[101] Nuyken O., Pask S.D. (2013). Ring-opening polymerization-An introductory review. Polymers.

[102] Kurzhals S., Gal N., Zirbs R., Reimhult E. (2017). Aggregation of thermoresponsive core-shell nanoparticles: Influence of particle concentration, dispersant molecular weight and grafting. J. Colloid Interface Sci..

[103] Popat A., Liu J., Lu G.Q., Qiao S.Z. (2012). A pH-responsive drug delivery system based on chitosan coated mesoporous silica nanoparticles. J. Mater. Chem..

[104] Yavuz M.S., Cheng Y., Chen J., Cobley C.M., Zhang Q., Rycenga M., Xie J., Kim C., Song K.H., Schwartz A.G. (2009). Gold nanocages covered by smart polymers for controlled release with near-infrared light. Nat. Mater..

[105] Han F., Soeriyadi A.H., Vivekchand S.R.C., Gooding J.J. (2016). Simple Method for Tuning the Optical Properties of Thermoresponsive Plasmonic Nanogels. ACS Macro Lett..

[106] Guardia P., Riedinger A., Nitti S., Pugliese G., Marras S., Genovese A., Materia M.E., Lefevre C., Manna L., Pellegrino T. (2014). One pot synthesis of monodisperse water soluble iron oxide nanocrystals with high values of the specific absorption rate. J. Mater. Chem. B.

[107] Xie S., Zhang B., Wang L., Wang J., Li X., Yang G., Gao F. (2015). Superparamagnetic iron oxide nanoparticles coated with different polymers and their MRI contrast effects in the mouse brains. Appl. Surf. Sci..

[108] Xu H.-L., Mao K.-L., Huang Y.-P., Yang J.-J., Xu J., Chen P.-P., Fan Z.-L., Zou S., Gao Z.-Z., Yin J.-Y. (2016). Glioma-targeted superparamagnetic iron oxide nanoparticles as drug-carrying vehicles for theranostic effects. Nanoscale.

[109] Chiu H., Haddick L., Engelke H., Bein T. (2018). Clickable Multifunctional Large-Pore Mesoporous Silica Nanoparticles as Nanocarriers. Chem. Mater..

[110] Brandenberger C., Mühlfeld C., Ali Z., Lenz A.G., Schmid O., Parak W.J., Gehr P., Rothen-Rutishauser B. (2010). Quantitative evaluation of cellular uptake and trafficking of plain and polyethylene glycol-coated gold nanoparticles. Small.

[111] Urries I., Muñoz C., Gomez L., Marquina C., Sebastian V., Arruebo M., Santamaria J. (2014). Magneto-plasmonic nanoparticles as theranostic platforms for magnetic resonance imaging, drug delivery and NIR hyperthermia applications. Nanoscale.

[112] Sun C., Du K., Fang C., Bhattarai N., Veiseh O., Kievit F., Stephen Z., Lee D., Ellenbogen R.G., Ratner B. (2010). PEG-Mediated Synthesis of Highly Dispersive Multifunctional Superparamagnetic Nanoparticles: Their Physicochemical Properties and Function In Vivo. ACS Nano.

[113] Wu L., Glebe U., Böker A. (2017). Synthesis of Polystyrene and Poly(4-vinylpyridine) Mixed Grafted Silica Nanoparticles via a Combination of ATRP and CuI-Catalyzed Azide-Alkyne Click Chemistry. Macromol. Rapid Commun..

[114] Tudisco C., Bertani F., Cambria M.T., Sinatra F., Fantechi E., Innocenti C., Sangregorio C., Dalcanale E., Condorelli G.G. (2013). Functionalization of PEGylated Fe_3_O_4_ magnetic nanoparticles with tetraphosphonate cavitand for biomedical application. Nanoscale.

[115] Das M., Bandyopadhyay D., Singh R., Harde H., Kumar S., Jain S. (2012). Orthogonal Biofunctionalization of Magnetic Nanoparticles via “Clickable” Poly-(Ethylene Glycol) Silanes: A “Universal Ligand” Strategy to Design Stealth and Target-Specific Nanocarriers. J. Mater. Chem..

[116] He H., Zhang Y., Gao C., Wu J. (2009). “Clicked” magnetic nanohybrids with a soft polymer interlayer. Chem. Commun..

[117] Khoee S., Bagheri Y., Hashemi A. (2015). Composition controlled synthesis of PCL–PEG Janus nanoparticles: Magnetite nanoparticles prepared from one-pot photo-click reaction. Nanoscale.

[118] Liu X., Cheng R., Deng J., Wu Y. (2014). Magnetic composite nanoparticles consisting of helical poly(n-hexyl isocyanate) and Fe3 O4 prepared via click reaction. RSC Adv..

[119] Oz Y., Arslan M., Gevrek T.N., Sanyal R., Sanyal A. (2016). Modular Fabrication of Polymer Brush Coated Magnetic Nanoparticles: Engineering the Interface for Targeted Cellular Imaging. ACS Appl. Mater. Interfaces.

[120] Hegazy M., Zhou P., Wu G., Wang L., Rahoui N., Taloub N., Huang X., Huang Y. (2017). Construction of polymer coated core–shell magnetic mesoporous silica nanoparticles with triple responsive drug delivery. Polym. Chem..

[121] Kabanov A.V., Vinogradov S.V. (2009). Nanogels as pharmaceutical carriers: Finite networks of infinite capabilities. Angew. Chem. Int. Ed..

[122] Sivaram A.J., Rajitha P., Maya S., Jayakumar R., Sabitha M. (2015). Nanogels for delivery, imaging and therapy. Wiley Interdiscip. Rev. Nanomed. Nanobiotechnol..

[123] Ferrer M.C.C., Dastgheyb S., Hickok N.J., Eckmann D.M., Composto R.J. (2014). Designing nanogel carriers for antibacterial applications. Acta Biomater..

[124] Tang Z., He C., Tian H., Ding J., Hsiao B.S., Chu B., Chen X. (2016). Polymeric nanostructured materials for biomedical applications. Prog. Polym. Sci..

[125] Wu H.Q., Wang C.C. (2016). Biodegradable smart nanogels: A new platform for targeting drug delivery and biomedical diagnostics. Langmuir.

[126] Molina M., Asadian-Birjand M., Balach J., Bergueiro J., Miceli E., Calderón M. (2015). Stimuli-responsive nanogel composites and their application in nanomedicine. Chem. Soc. Rev..

[127] Thoniyot P., Tan M.J., Karim A.A., Young D.J., Loh X.J. (2015). Nanoparticle-Hydrogel Composites: Concept, Design, and Applications of These Promising, Multi-Functional Materials. Adv. Sci..

[128] Cao Z.Q., Wang G.J. (2016). Multi-Stimuli-Responsive Polymer Materials: Particles, Films, and Bulk Gels. Chem. Rec..

[129] Sierra-Martin A., Fernandez-Barbero B. (2015). Multifunctional hybrid nanogels for theranostic applications. Soft Matter.

[130] Wu W., Zhou S. (2013). Responsive Polymer-Inorganic Hybrid Nanogels for Optical Sensing, Imaging, and Drug Delivery. Nanomater. Drug Deliv. Imaging Tissue Eng..

[131] Liu J., Detrembleur C., Mornet S., Jérôme C., Duguet E. (2015). Design of hybrid nanovehicles for remotely triggered drug release: An overview. J. Mater. Chem. B.

[132] Ryu J.H., Koo H., Sun I.C., Yuk S.H., Choi K., Kim K., Kwon I.C. (2012). Tumor-targeting multi-functional nanoparticles for theragnosis: New paradigm for cancer therapy. Adv. Drug Deliv. Rev..

[133] Merino S., Martín C., Kostarelos K., Prato M., Vázquez E. (2015). Nanocomposite hydrogels: 3D polymer-nanoparticle synergies for on-demand drug delivery. ACS Nano.

[134] Yang J., Yao M., Wen L., Song J., Zhang M., Zhao Y., Liu B. (2014). Multifunctional quantum dot-polypeptide hybrid nanogel for targeted imaging and drug delivery. Nanoscale.

[135] Khatun Z., Nurunnabi M., Nafiujjaman M., Reeck G.R., Khan H.A., Cho K.J., Lee Y. (2015). A hyaluronic acid nanogel for photo–chemo theranostics of lung cancer with simultaneous light-responsive controlled release of doxorubicin. Nanoscale.

[136] Wang H., Di J., Sun Y., Fu J., Wei Z., Matsui H., Del A., Alonso C., Zhou S. (2015). Biocompatible PEG-Chitosan@Carbon Dots Hybrid Nanogels for Two-Photon Fluorescence Imaging, Near-Infrared Light/pH Dual-Responsive Drug Carrier, and Synergistic Therapy. Adv. Funct. Mater..

[137] Wu W., Mitra N., Yan E.C.Y., Zhou S. (2010). Multifunctional Hybrid Nanogel for and Self-Regulated Insulin Release at Physiological pH. ACS Nano.

[138] Maya S., Sarmento B., Nair A., Rejinold N.S., Nair S.V., Jayakumar R. (2013). Smart stimuli sensitive nanogels in cancer drug delivery and imaging: A review. Curr. Pharm. Des..

[139] Kowalczuk A., Trzcinska R., Trzebicka B., Müller A.H.E., Dworak A., Tsvetanov C.B. (2014). Loading of polymer nanocarriers: Factors, mechanisms and applications. Prog. Polym. Sci..

[140] Chen T., Cao Z., Guo X., Nie J., Xu J., Fan Z., Du B. (2011). Preparation and characterization of thermosensitive organic–inorganic hybrid microgels with functional Fe_3_O_4_ nanoparticles as crosslinker. Polymer.

[141] Messing R., Frickel N., Belkoura L., Strey R., Rahn H., Odenbach S., Schmidt A.M. (2011). Cobalt ferrite nanoparticles as multifunctional cross-linkers in PAAm ferrohydrogels. Macromolecules.

[142] Zhou A., Luo H., Wang Q., Chen L., Zhang T.C., Tao T. (2015). Magnetic thermoresponsive ionic nanogels as novel draw agents in forward osmosis. RSC Adv..

[143] Socoliuc V., Vékás L., Turcu R. (2013). Magnetically induced phase condensation in an aqueous dispersion of magnetic nanogels. Soft Matter.

[144] Liu J., Detrembleur C., Debuigne A., de Pauw-Gillet M.-C., Mornet S., Elst L.V., Laurent S., Duguet E., Jerome C. (2014). Glucose-, pH- and thermo-responsive nanogels crosslinked by functional superparamagnetic maghemite nanoparticles as innovative drug delivery systems. J. Mater. Chem. B.

[145] Sun H., Yu J., Gong P., Xu D., Zhang C., Yao S. (2005). Novel core–shell magnetic nanogels synthesized in an emulsion-free aqueous system under UV irradiation for targeted radiopharmaceutical applications. J. Magn. Magn. Mater..

[146] Demarchi C.A., Debrassi A., Buzzi F.D.C., Corrêa R., Filho V.C., Rodrigues C.A., Nedelko N., Demchenko P., Ślawska-Waniewska A., Dłużewski P. (2014). A magnetic nanogel based on *O*-carboxymethylchitosan for antitumor drug delivery: Synthesis, characterization and in vitro drug release. Soft Matter.

[147] Wang X., Niu D., Li P., Wu Q., Bo X., Liu B., Bao S., Su T., Xu H., Wang Q. (2015). Dual-Enzyme-Loaded Multifunctional Hybrid Nanogel System for Pathological Responsive Ultrasound Imaging and T2-Weighted Magnetic Resonance Imaging. ACS Nano.

[148] Wang H., Ke F., Mararenko A., Wei Z., Banerjee P., Zhou S. (2014). Responsive polymer–fluorescent carbon nanoparticle hybrid nanogels for optical temperature sensing, near-infrared light- responsive drug release, and tumor cell imaging. Nanoscale.

[149] Liu G., Cai M., Wang X., Zhou F., Liu W. (2016). Magnetite-Loaded Thermosensitive Nanogels for Bioinspired Lubrication and Multimodal Friction Control. ACS Macro Lett..

[150] Jiang L., Zhou Q., Mu K., Xie H., Zhu Y., Zhu W., Zhao Y., Xu H., Yang X. (2013). PH/temperature sensitive magnetic nanogels conjugated with Cy5.5-labled lactoferrin for MR and fluorescence imaging of glioma in rats. Biomaterials.

[151] Zhou Y.T., Nie H.-L., Branford-White C., He Z.-Y., Zhu L.M. (2009). Removal of Cu^2+^ from aqueous solution by chitosan-coated magnetic nanoparticles modified with α-ketoglutaric acid. J. Colloid Interface Sci..

[152] Yang J., Yao M.H., Jin R.M., Zhao D.H., Zhao Y., Liu B. (2017). Polypeptide-Engineered Hydrogel Coated Gold Nanorods for Targeted Drug Delivery and Chemo-photothermal Therapy. ACS Biomater. Sci. Eng..

[153] Boularas M., Gombart E., Tranchant J.-F., Billon L., Save M. (2015). Design of Smart Oligo(ethylene glycol)-Based Biocompatible Hybrid Microgels Loaded with Magnetic Nanoparticles. Macromol. Rapid Commun..

[154] Boularas M., Deniau-Lejeune E., Alard V., Tranchant J.-F. (2015). Maud Save, Laurent Billon, Dual stimuli-responsive oligo(ethylene glycol)-based microgels: Insight into the role of internal structure in volume phase transitions and loading of magnetic nanoparticles to design stable thermoresponsive hybrid microgels. Polym. Chem..

[155] Cazares-Cortes E., Espinosa A., Guigner J.M., Michel A., Griffete N., Wilhelm C., Ménager C. (2017). Doxorubicin Intracellular Remote Release from Biocompatible Oligo(ethylene glycol) Methyl Ether Methacrylate-Based Magnetic Nanogels Triggered by Magnetic Hyperthermia. ACS Appl. Mater. Interfaces.

[156] Chiang W.H., Ho V.T., Chen H.H., Huang W.C., Huang Y.F., Lin S.C., Chern C.S., Chiu H.C. (2013). Superparamagnetic hollow hybrid nanogels as a potential guidable vehicle system of stimuli-mediated MR imaging and multiple cancer therapeutics. Langmuir.

[157] Rajar K., Karakus B., Koc K., Alveroglu E. (2016). One pot synthesis and characterization of Fe3O4 Nanorod-PNIPA Nanogel Composite for protein adsorption. Mater. Sci. Eng. C.

[158] Chowdhury P., Kr S., Guha A., Kr S. (2012). Chemical and biochemical activities of sonochemically synthesized poly (*N*-isopropyl acrylamide)/silica nanocomposite. Appl. Surf. Sci..

[159] Khaled S.Z., Cevenini A., Yazdi I.K., Parodi A., Evangelopoulos M., Corbo C., Scaria S., Hu Y., Haddix S.G., Corradetti B. (2016). One-pot synthesis of pH-responsive hybrid nanogel particles for the intracellular delivery of small interfering RNA. Biomaterials.

[160] Schoth A., Adurahim E.S., Bahattab M.A., Landfester K., Muñoz-Espí R. (2016). Waterborne Polymer/Silica Hybrid Nanoparticles and Their Structure in Coatings. Macromol. React. Eng..

[161] Schoth A., Keith A.D., Landfester K., Muñoz-Espí R. (2016). Silanization as a versatile functionalization method for the synthesis of polymer/magnetite hybrid nanoparticles with controlled structure. RSC Adv..

[162] Fu G.D., Jiang H., Yao F., Xu L.Q., Ling J., Kang E.T. (2012). Preparation of fluorescent organometallic porphyrin complex nanogels of controlled molecular structure via reverse-emulsion click chemistry. Macromol. Rapid Commun..

[163] Soleimani K., Tehrani A.D.D., Adeli M. (2018). Bioconjugated graphene oxide hydrogel as an effective adsorbent for cationic dyes removal. Ecotoxicol. Environ. Saf..

[164] Asadian-Birjand M., Biglione C., Bergueiro J., Cappelletti A., Rahane C., Chate G., Khandare J., Klemke B., Strumia M.C., Calderón M. (2016). Transferrin decorated thermoresponsive nanogels as magnetic trap devices for circulating tumor cells. Macromol. Rapid Commun..

[165] Biglione C., Bergueiro J., Asadian-Birjand M., Weise C., Khobragade V., Chate G., Dongare M., Khandare J., Strumia M.C., Calderón M. (2018). Optimizing circulating tumor cells’ capture efficiency of magnetic nanogels by transferrin decoration. Polymers.

[166] Anirudhan T.S., Sandeep S. (2012). Synthesis, characterization, cellular uptake and cytotoxicity of a multi-functional magnetic nanocomposite for the targeted delivery and controlled release of doxorubicin to cancer cells. J. Mater. Chem..

[167] Yuan L., Tang Q., Yang D., Zhang J.Z., Zhang F., Hu J. (2011). Preparation of pH-Responsive Mesoporous Silica Nanoparticles and Their Application in Controlled Drug Delivery. J. Phys. Chem. C.

[168] Sun L., Wang D., Chen Y., Wang L., Huang P., Li Y., Liu Z., Yao H., Shi J. (2017). Core-shell hierarchical mesostructured silica nanoparticles for gene/chemo-synergetic stepwise therapy of multidrug-resistant cancer. Biomaterials.

[169] Calderón M., Quadir M.A., Strumia M., Haag R. (2010). Functional dendritic polymer architectures as stimuli-responsive nanocarriers. Biochimie.

[170] Paez J.I., Martinelli M., Brunetti V., Strumia M.C. (2012). Dendronization: A useful synthetic strategy to prepare multifunctional materials. Polymers.

[171] Brunetti V., Bouchet L.M., Strumia M.C. (2015). Nanoparticle-cored dendrimers: Functional hybrid nanocomposites as a new platform for drug delivery systems. Nanoscale.

[172] Gao J., Jiang Y., Lu J., Han Z., Deng J., Chen Y. (2017). Dopamine-functionalized mesoporous onion-like silica as a new matrix for immobilization of lipase *Candida* sp. 99–125. Sci. Rep..

[173] Botella P., Abasolo I., Fernández Y., Muniesa C., Miranda S. (2011). Surface-modified silica nanoparticles for tumor-targeted delivery of camptothecin and its biological evaluation. J. Control. Release.

[174] Maggini L., Cabrera I., Ruiz-Carretero A., Prasetyanto E.A., Robinet E., de Cola L. (2016). Breakable mesoporous silica nanoparticles for targeted drug delivery. Nanoscale.

[175] Contreras-Cáceres R., Pacifico J., Pastoriza-Santos I., Pérez-Juste J., Fernández-Barbero A., Liz-Marzán L.M. (2009). Au@pNIPAM thermosensitive nanostructures: Control over shell cross-linking, overall dimensions, and core growth. Adv. Funct. Mater..

[176] Qiao L., Wang X., Gao Y., Wei Q., Hu W., Wu L., Li P., Zhu R., Wang Q. (2016). Laccase-mediated formation of mesoporous silica nanoparticle based redox stimuli-responsive hybrid nanogels as a multifunctional nanotheranostic agent. Nanoscale.

[177] Wang Y., Wang L., Yan M., Dong S., Hao J. (2017). Near-Infrared-Light-Responsive Magnetic DNA Microgels for Photon- and Magneto-Manipulated Cancer Therapy. ACS Appl. Mater. Interfaces.

[178] Vicario-de-la-Torre M., Forcada J. (2017). The Potential of Stimuli-Responsive Nanogels in Drug and Active Molecule Delivery for Targeted Therapy. Gels.

[179] Zhang W., Liu M., Liu A., Zhai G. (2017). Advances in Functionalized Mesoporous Silica Nanoparticles for Tumor Targeted Drug Delivery. Curr. Pharm. Sci..

[180] Yan J., Pan X., Schmitt M., Wang Z., Bockstaller M.R., Matyjaszewski K. (2016). Enhancing Initiation Efficiency in Metal-Free Surface-Initiated Atom Transfer Radical Polymerization (SI-ATRP). ACS Macro Lett..

[181] Aydogan C., Yilmaz G., Yagci Y. (2017). Synthesis of Hyperbranched Polymers by Photoinduced Metal-Free ATRP. Macromolecules.

[182] Zhou H., Wang X., Tang J., Yang Y.-W. (2016). Surface Immobilization of pH-Responsive Polymer Brushes on Mesoporous Silica Nanoparticles by Enzyme Mimetic Catalytic ATRP for Controlled. Polymers.

